# Homologous Recombination Deficiency in Ovarian, Breast, Colorectal, Pancreatic, Non-Small Cell Lung and Prostate Cancers, and the Mechanisms of Resistance to PARP Inhibitors

**DOI:** 10.3389/fonc.2022.880643

**Published:** 2022-06-17

**Authors:** Negesse Mekonnen, Hobin Yang, Young Kee Shin

**Affiliations:** ^1^ Department of Pharmacy, Research Institute of Pharmaceutical Science, Seoul National University College of Pharmacy, Seoul, South Korea; ^2^ Department of Veterinary Science, School of Animal Science and Veterinary Medicine, Bahir Dar University, Bahir Dar, Ethiopia; ^3^ Bio-MAX/N-Bio, Seoul National University, Seoul, South Korea; ^4^ Department of Molecular Medicine and Biopharmaceutical Sciences, Seoul National University Graduate School of Convergence Science and Technology, Seoul, South Korea; ^5^ LOGONE Bio Convergence Research Foundation, Center for Companion Diagnostics, Seoul, South Korea

**Keywords:** pathogenic mutation, loss of heterozygosity, promoter hypermethylation, DNA repair genes, hereditary and familial cancer, PARP inhibitor, base excision repair

## Abstract

Homologous recombination (HR) is a highly conserved DNA repair mechanism that protects cells from exogenous and endogenous DNA damage. Breast cancer 1 (BRCA1) and breast cancer 2 (BRCA2) play an important role in the HR repair pathway by interacting with other DNA repair proteins such as Fanconi anemia (FA) proteins, ATM, RAD51, PALB2, MRE11A, RAD50, and NBN. These pathways are frequently aberrant in cancer, leading to the accumulation of DNA damage and genomic instability known as homologous recombination deficiency (HRD). HRD can be caused by chromosomal and subchromosomal aberrations, as well as by epigenetic inactivation of tumor suppressor gene promoters. Deficiency in one or more HR genes increases the risk of many malignancies. Another conserved mechanism involved in the repair of DNA single-strand breaks (SSBs) is base excision repair, in which poly (ADP-ribose) polymerase (PARP) enzymes play an important role. PARP inhibitors (PARPIs) convert SSBs to more cytotoxic double-strand breaks, which are repaired in HR-proficient cells, but remain unrepaired in HRD. The blockade of both HR and base excision repair pathways is the basis of PARPI therapy. The use of PARPIs can be expanded to sporadic cancers displaying the “BRCAness” phenotype. Although PARPIs are effective in many cancers, their efficacy is limited by the development of resistance. In this review, we summarize the prevalence of HRD due to mutation, loss of heterozygosity, and promoter hypermethylation of 35 DNA repair genes in ovarian, breast, colorectal, pancreatic, non-small cell lung cancer, and prostate cancer. The underlying mechanisms and strategies to overcome PARPI resistance are also discussed.

## 1 Introduction

Homologous recombination (HR) is one of the major pathways for the repair of DNA double-strand breaks (DSBs) in eukaryotic cells. Pathogenic mutations in genes encoding HR-related proteins are associated with the development of certain malignancies, including breast, ovarian, and other cancers (1). Normal and cancer cells rely on multiple DNA damage response pathways that specifically repair different forms of DNA damage. Key pathways include homologous recombination repair (HRR), base excision repair (BER), nucleotide excision repair (NER), mismatch repair (MMR), nonhomologous end-joining (NHEJ), translesion synthesis (TLS), and interstrand crosslink (ICL) repair (2, 3). However, these repair pathways are not equally effective in DNA repair, and some mechanisms are error-prone. For instance, non-canonical DNA repair systems such as NHEJ, single-strand annealing, and TLS are activated when the canonical pathways are deficient (4, 5). In response to DSBs, cells activate the HRR pathway, which relies on the undamaged sister chromatid as a template for repair. Because of this reliance on sister chromatids, HRR is active during the S and G2 phases of the cell cycle and is a high fidelity and error-free DNA repair pathway (3). By contrast, in NHEJ, the break ends are directly ligated without a homologous template, resulting in an error-prone repair pathway that can predispose to genetic instability (6, 7).

Genomic scars in relation to HR are caused by chromosomal and sub chromosomal aberrations. Genomic aberrations arise from mutation, structural copy number aberrations, or structural rearrangements. Mutations are substitutions (transversion & transition) or indel (insertion & deletion) mutations, and can inactivate tumor suppressor genes (TSGs). Structural copy number alterations can be copy number gain (leading to allelic imbalance) or copy number loss [leading to deletion, loss of heterozygosity (LOH), or haplo-insufficiency (one copy of a gene is deleted or contain loss of function mutation leading to insufficient level of proteins)]. Structural rearrangements can be inversion (paracentric), translocation (reciprocal), or recombination leading to copy neutral LOH events (8). Frequent copy number alterations are the hallmarks of homologous recombination deficiency (HRD) and can occur at the regional or whole chromosome level. Quantification of large-scale structural variants is used as an indicator of the HRD phenotype (presence of HRD in sporadic cancers other than BRCA1 and 2 inactivation), including telomeric allelic imbalance (TAI: large allelic imbalances extending to the telomere), large scale transition (LST: number of transitions between large regions of different allelic states or chromosomal breaks between adjacent regions of >10 MB), and LOH (large regions displaying somatic loss of one haplotype, which can be copy variable as in deletion or copy neutral LOH). Genes involved in HR, including tumor suppressor genes, can also be repressed by aberrant promoter hypermethylation, an epigenetic mechanism that contributes to HRD (9).

HR is a DNA repair pathway of clinical interest because of the sensitivity of HRD cells to poly-ADP-ribose polymerase (PARP) inhibitors (PARPIs) (10). DNA repair targeting therapies exploit DNA repair defects in cancer cells to generate synthetic lethality, and DNA repair defects vary according to cancer type. For example, approximately 50% of ovarian carcinomas exhibit dysfunctional HRR, whereas the rate of HRR dysfunction is lower in other cancer types such as colorectal cancer (CRC) (<5%) (11, 12). Hereditary mutations in one copy of the *BRCA* gene predispose patients to female breast cancer (85% lifetime risk), ovarian cancer (10%–40%), male breast cancer, pancreatic cancer (PC), prostate cancer, non-small cell lung cancer (NSCLC), CRC, and other cancer types. Precancerous cells deficient in BRCA1 and 2 cannot repair DSBs properly, resulting in genomic instability that eventually leads to cancer (8). These tumors are intrinsically sensitive to DNA damage response inhibitors (PARPIs), which induce synthetic lethality. Synthetic lethality can arise from the combined inactivation of HRR genes by (mutation, LOH and promoter hypermethylation) and PARP inhibition (13). The use of PARPIs in non-*BRCA* mutation carrier patients can be expanded to sporadic cancers that display “BRCAness” (cancers that have defective HR without germline *BRCA1* and *2* mutations). Findings showed that TSGs with BRCAness phenotypes are often inactivated, for example, *ATM*, *ATR*, *PALB2*, *RAD51*, *RAD51B*, *RAD51C*, *RAD51D*, *BARD1*, and *FANCM* among others (14–17).

The use of PARPIs was recently expanded to other cancers in addition to breast and ovarian cancer, such as prostate and PC (18). Although PARPIs have shown beneficial effects in many other cancer types, the frequent development of resistance is challenging. For instance, in a phase II clinical trial, secondary resistance mutations were detected in circulating free tumor DNA in two patients with a germline *BRCA2* mutation. These mutations were predicted to lead to the reversal of a somatic mutation (19). A comprehensive investigation of the underlying mechanisms is necessary to design strategies for overcoming PARPI resistance.

Another challenging issue is the development of effective biomarkers to identify patients who are more likely to respond to specific targeted therapy by using companion diagnosis (CDx). CDx is an *in vitro* medical device that uses biomarkers to provide information on the safe and effective use of drugs or biologicals. FDA-approved CDx includes BRACAnalysis CDx^®^ and Myriad myChoice^®^ CDx developed by Myriad Genetic Laboratories, and FoundationOne^®^ CDx [F1CDx] and FoundationOne^®^ Liquid CDx developed by Foundation medicine. The HRDetect test utilizes machine learning algorithm (20).

BRACAnalysis CDx^®^ is an *in vitro* diagnostic method used for the detection and classification of DNA sequence variants in the protein-coding regions, intron or exon boundaries of the germline *BRCA1* and *2* genes from whole blood sample. PCR and Sanger sequencing are used to detect small insertions and deletions (indels), and single nucleotide variants (SNVs). Large deletions and duplications are detected by multiplex PCR. The test results used as an aid to identify eligible patients for PARPIs in breast, ovarian, pancreatic and prostate cancers treatment (21). Myriad myChoice^®^ CDx is NGS-based *in vitro* diagnostic test that evaluates the qualitative detection and classification of SNVs, indels and large rearrangements (LRs) in protein-coding regions and intron/exon boundaries of the *BRCA1* and *2* genes, and determine Genomic Instability Score (GIS) by measuring [LOH, TAI, and LST] using DNA isolated from formalin-fixed paraffin-embedded (FFPE) tumor tissue. The test used to select eligible patients for ovarian cancer with positive HRD status for the treatment with Zejula^®^ (niraparib) (22).

FoundationOne^®^CDx (F1CDx) is a qualitative NGS- and high throughput hybridization-based capture test for the detection of indels, substitutions and copy number alterations (CNAs) in 324 genes. It detects gene rearrangements, genomic signatures including microsatellite instability (MSI), tumor mutational burden (TMB) and positive HRD status (somatic BRCA-positive and/or LOH high) using DNA isolated from FFPE tumor tissue. It provides definite information for the identification of eligible patients for specific treatments of different class using specific biomarkers for many solid tumors (23).

FoundationOne^®^ Liquid CDx is a qualitative NGS based test, which can identify indels, and substitutions in 311 genes, rearrangements in 4 genes and CNAs in 3 genes. It utilizes circulating cell-free DNA (cfDNA) isolated from plasma-driven peripheral whole blood collected in anti-coagulants. The test identifies patients that can benefit from different targeted treatments for NSCLC, breast, ovarian and prostate cancers based on specific biomarkers detected in each cancer. Negative result does not rule out the presence of an alteration in the patient’s tumor, in this case patients can opt for another tumor tissue-based CDx. The test analytical accuracy is not well demonstrated in all genes e.g., the test does not detect heterozygous deletions, and copy number losses/homozygous deletion in ATM (24).

HRDetect is a whole genome sequencing (WGS)-based classifier of HRD that can predict *BRCA1* and *2* deficiency based on six mutational signatures (the HRD index [LOH + TAI + LST], microhomology-mediated indels, base-substitution signature 3 and 8, and rearrangement signature 3 and 5). It can also identify HRD in sporadic cancers (BRCAness) with and without any single detectable defect in HR genes (20, 25). HRDetect was shown highly sensitive method as compared to other HRD detection CDxs (20), but require clinical validation in independent set to avoid overfitting issue.

In this review, we did not classify mutation and hypermethylation data as bi-allelic or mono-allelic inactivation. Oftentimes, cases of pathogenic mutation in tumor suppressor genes lead to bi-allelic inactivation. Whole-exome sequencing analysis of breast cancer cases by Mutter et al. (26) revealed that 89% of bi-allelic inactivation results in HRD, whereas in cases of mono-allelic inactivation significant association existed between RAD51 functional status and LST. In a study by Li et al. (27) mono-allelic germline pathogenic mutation of *PALB2* had predisposed to a high-risk breast cancer development, underscoring the role of PALB2 in HR repair. Moreover, protein-truncating variants and rare missense variants of DNA repair genes were significantly associated with the risk of breast cancer (16). Many findings confirmed the importance of haplo-insufficiency in tissue and gene specific manner; for instance, PTEN hypermorphic mice expressing 80% normal levels of PTEN protein was sufficient to predispose for different cancers development (28). Mono-allelic inactivation of TSGs (e.g., p53 and PTEN) leads to the inability to perform normal cellular functions which contributed to cancer development (29).

Here, we investigated the potential implications of pathogenic mutations, LOH, and promoter hypermethylation of HR-related genes using recent data in ovarian, breast, colorectal, pancreatic, non-small cell lung, and prostate cancers. Additionally, the mechanisms underlying PARPI resistance and possible strategies to overcome PARPI resistance are discussed.

### 1.1 BRCA1 and BRCA2 in Homologous Recombination Repair

BRCA1 and 2 interacts with a number of other DNA repair proteins to form a complex system for DNA damage repair, including ATM, RAD51, PALB2, MRE11A, RAD50, NBN, and the Fanconi anemia proteins (30). BRCA1 and BRCA2 are potential biomarkers for HRD in ovarian and breast cancer. In the presence of DNA DSBs, BRCA1 and 2 collaborate with other HR proteins to maintain the breaks. For instance, ATM is specifically activated in response to DSBs and is essential for phosphorylating many proteins involved in controlling cell cycle checkpoints and DNA repair. Three proteins are involved in recruiting ATM to DSBs, meiotic recombination 11 (MRE11A), RAD50, and NBS1 or MRN complex. Cells deficient in ATM and NBS1 are thus sensitive to PARPIs, similar to BRCA1- and BRCA2-deficient cells (7, 31). Germline pathogenic mutations of *BRCA1* and *BRCA2* suppress the HR mechanism and cause hereditary breast and ovarian cancer (HBOC) syndrome (32, 33). The functions of 35 HR-related genes are described briefly in **Supplementary Table S1**.

### 1. 2 Interaction Between *FANC* and *BRCA* Genes in Homologous Recombination Repair

**Figure 1:**
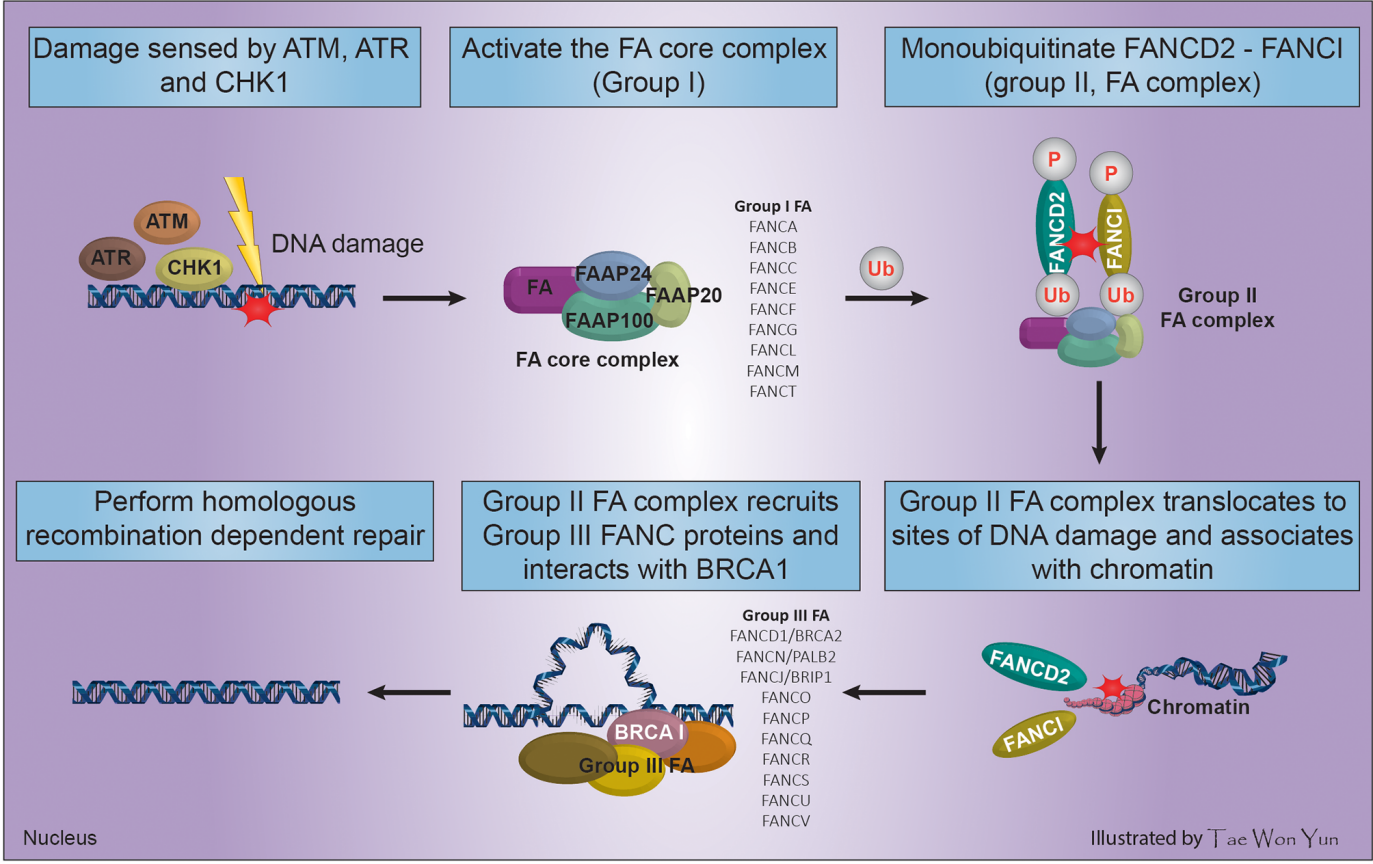
Process of FA complex formation and DNA interstrand cross-linking maintenance through the interaction of *FANC* and *BRCA* genes.

Fanconi anemia (FA) is a clinically and genetically heterogeneous syndrome involving bone marrow failure (BMF), developmental/congenital abnormalities that may affect all organ systems (renal dysplasia, craniofacial malformations, endocrine dysfunction, developmental delay, VACTERL association, radial ray malformations, osteoporosis, progressive BMF, skin abnormalities, short stature, cardiac defects, decreased fertility, and genitourinary and gastrointestinal malformations), and cancer predisposition (34). This review focused only on the role of five FA genes in cancer predisposition. FA is a congenital defect that results from loss of function of any of 21 genes, which indicates their essential role in maintaining the chromosomal stability of hematopoietic stem cells. The main cause of FA Complementation Group (*FANC*) gene abnormality is mutation (95%). The unique clinical phenotype associated with *FANC* gene mutations implies that proteins encoded by these genes function in a common cellular pathway. This pathway, known as the FA/BRCA DNA repair pathway (**Figure 1**), preserves genomic homeostasis in response to specific types of DNA damage (35). The FA pathway serves to remove ICLs and shares components, such as BRCA2 and PALB2, with the HR and NER pathways (2).

The main genome housekeeping function of the FA pathway in the DNA damage response necessitates multifactorial activation of HR. Cells use the error-prone NHEJ repair mechanism when FA-based repair systems are deficient, which may negatively affect genomic stability (36). In response to DNA damage caused by radiation, tobacco smoke, alcohol, or reactive oxygen species among others, FA proteins and three other Fanconi-associated proteins (FAAP100, FAAP24, and FAAP20) are activated by DNA damage response sensors such as ATM, ATR, and cell cycle check point (CHK1) to arrest the cell cycle, forming the FA core complex (group I) (34). The assembled FA core complex (group I) binds to the ubiquitin-conjugating enzyme UBE2T *via* the FANCL subunit and activates the FANCD2-FANCI complex (group II FA complex) through mono-ubiquitination and phosphorylation of FANCD2/FANCI (2). The ubiquitinated FANCD2-FANCI complex translocates to sites of DNA damage, associates with chromatin, and co-localizes with/recruits the downstream FA effector proteins (group III FA complex) including FANCD1/BRCA2, FANCN/PALB2, and FANCJ/BRIP1. Then, the group III FA complex perform HR-dependent DNA repair by interacting with BRCA1 (**Figure 1**) (35, 37).

## 2 Homologous Recombination Deficiency in Ovarian Cancer

The International Agency for Research on Cancer (IARC) (38) reports that ovarian cancer is the 8^th^ and 9^th^ cause of incidence and mortality worldwide in females, respectively. In 2020, the incidence and mortality of ovarian cancer were estimated at 3.4% (313,959 of all cancer types) and 4.68% (207,252 of all cancer types), respectively. Approximately 75% of epithelial ovarian cancer patients are diagnosed with advanced disease, which is curable in only a minority of cases, resulting in a modest 5-year survival rate of 20–30% (39). According to the cancer genome atlas (TCGA), high grade serous ovarian cancers (HGSOCs) are characterized by frequent genetic and epigenetic alterations of HR pathway genes, most commonly *BRCA1* and *BRCA2*. In addition, approximately 50% of patients with HGSOC exhibit genetic and epigenetic alterations in the FANC-BRCA (**Figure 1**) pathway (11). Germline mutations in the *BRCA1* and *BRCA2* genes are well-known mechanisms of HRD, and loss of BRCA1 or BRCA2 thus poses a significant risk to genome integrity, leading not only to cancer predisposition, but also affecting the sensitivity to DNA-damaging agents and thus therapeutic approaches (40). Pathogenic variants of *BRCA1* and *BRCA2* only explain the genetic cause of approximately 10% of hereditary breast and ovarian cancers (transmitted to offspring), underscoring the clinical importance of testing other DNA repair genes (41). Findings showed that BRCA1 and 2 inactivation frequently led to higher HRD score in ovarian and breast cancers. This high HRD score has positive prognostic significance for platinum and PARPI therapy. In unclassified ovarian cancer patients who undertook germline *BRCA1* and *2* test, 19% (44/235) were carriers of germline mutations, and somatic mutation test was done on 28 specimens, 42.9% (9/21) and 28.6% (2/7) were found to be *BRCA1* and *2* positives, respectively (42). In another study by Pennington et al. (43), among 367 ovarian carcinomas tested for somatic mutation, 2.5% (19/367) and 1.63% (6/367) were positive for *BRCA1* and *2*, respectively. These carriers of somatic mutation have shown a positive impact on overall survival and platinum responsiveness as germline *BRCA1* and *2* mutation carriers. Other factors such as germline and somatic mutations in HR genes (**Table 1**) and epigenetic alterations (promoter hypermethylation) are implicated in HRD (84).

**Table 1:** Prevalence of mutation, LOH, and promoter hypermethylation in ovarian cancer.

Genes	Mutation [%(proportion)]	LOH [%(proportion)]	Promoter Methylation [%(proportion)]
**BRCA1**	12.2% (31/255) (44); 5% (15/300) (45); 18% (60/333) (46); 15.5% (81/523) (47); 16.5% (26/158) (48)	88% (36/41) (49); 44% (4/9) (50); 67% (6/9) (50); 81.5% (123/151) (51); 60% (60/100) (52); 97% (30/31) (53); 10.13% (16/158) (48)	20% (22/112) (54); 14% (2/14) (50); 14% (5/35) (55); 9.6% (32/332) (56); 14% (38/257) (57); 35% (15/42) (58); 9.34% (45/482) (59); 73.7% (56/76) (60)
**BRCA2**	9.8% (25/255) (44); 2% (6/300) (45); 3.3% (11/333) (46); 5.5% (29/523) (47); 5.06% (8/158) (48)	58% (24/41) (49); 50% (3/6) (50); 67% (4/6)] (50); 68.9% (104/151) (51); 73% (75/103) (52); 53% (16.5/31) (53); 0.63% (1/158) (48)	21% (3/14) (50); 44% (22/50) (61)
**RAD50**	7.7% (29/380) (62); 60% (12/20) (63); 2.94% (2/68) (64); 0.63% (1/158) (48)	0.63% (1/158) (48)	–
**RAD51**	0.3% (1/316) (11)	2% (10/489) (11)	–
**RAD51B**	2.1% (3/142) (65); 0.06% (2/3.429) (66)	0.8% (4/489) (11)	–
**RAD51C**	0.7% (1/141) (67); 2.5% (13/523) (47); 0.41% (14/3,429) (66)	97% (30/31) (53); 0.5 (2/429) (68)	1.45% (7/482) (59); 2.7% (9/332) (69); 2.67% (14/524) (70); 3% (9/316) (11)
**RAD51D**	1.3% (1/77) (67); 2.6% (10/380) (62); 0.35% (12/3429) (66)	0.7% (3/429) (68); 1.2% (6/489) (11)	–
**PALB2**	3% (9/299) (71); 3.03% (2/66) (72); 0.6% (2/333) (46); 0.63% (12/1915) (73); 2.9% (2/69) (74); 1.1% (6/523) (47); 1.9% (3/158) (48)	0.23% (1/429) (75); 0.7% (3/429) (68); 10.8% (17/158) (48)	3.08% (4/130) (76)
**FANCA**	4.35% (1/23) (45)	56.45% (17.5/31) (53); 1.16% (1/86) (77); 0.7% (3/429) (75)	–
**FANCD2**	0.3% (1/316) (11)	32.25% (10/31) (53); 0.23% (1/429) (75)	–
**FANCF**	0.3% (1/300) (45)	0.2% (1/572) (78)	32.14% (36/112) (79);13.2% (7/53) (80)
**FANCI**	0.6% (92/300) (45)	1.16% (1/86) (77)	–
**FANCM**	4.35% (1/23) (45); 2.1% (5/235) (81); 0.96% (5/523) (47)	0.2% (1/489) (11)	–
**NBN/NBS1**	1.8% (6/333) (46); 0.28% (9/3236) (82); 0.42% (1/235) (81); 0.38% (2/523) (47)	0.6% (3/489) (11)	–
**BARD1**	0.12% (4/3,236) (82); 1.6% (4/255) (83); 0.63% (1/158) (48)	0.63% (1/158) (48)	–
**ATM**	1.78% (7/392) (121); 0.3% (1/333) (46); 16.7% (8/48) (292); 0.82% (3/367 (43); 3.2% (5/158) (48)	29% (9/31) (53); 1.86% (8/429) (75); 1.9% (3/158) (48)	–
**ATR**	6% (3/50) (293); 69.7% (23/33) (294);; 4.8% (12/141) (295)	29% (9/31) (53) (75);	–
**MRE11A**	5.92% (17/287) (296); 0.4% (2/523) (47); 0.22% (1/466) (297)		–
**BRIP1**	7.7% (29/380) (62); 1.47% (1/68) (64); 0.4% (2/523) (47); 1.7% (8/466) (297); 0.52% (1/192) (131); 0.63% (1/158) (48)	0.7% (3/429) (68); 1.3% (2/158) (48)	–
**ERCC1**	2.6% 10/380) (62); 0.2% (1/523) (78)	0.4% (2/489) (11)	–
**CHEK2**	20.3% (77/380) (62); 45% (9/20) (63); 1.47% (1/68) (64); 4.2% (12/287) (296); 0.4% (2/523) (47); 1.72% (10/581) (298); 0.43% (2/466) (297); 0.52% (1/192) (131); 0.63% (1/158) (48)	10% (1/10) (298); 7.6% (12/158) (48)	–
**EMSY**	3.8% (14/380) (62); 8% (25/316) (11); 1.5% (8/523) (78)	0.2% (1/489) (11)	–
**TP53**	1.47% (1/68) (64); 3.83% (11/287) (296); 0.3% (2/581) (298); 1.04% (2/192) (131); 96% (312/316) (11); 57% (90/158) (48); 71.3% (375/523) (78)	0.63% (1/158) (78)	–
**STK11**	4.2% (12/287) (296); 1.3% (2/158) (48)	1.6% (8/489) (11)	–
**PTEN**	5.23% (15/287) (296); 0.43% (2/466) (297); 11.4% (18/158) (48)	6.7% (21/316) (11); 1.9% (3/158) (48); 6.1% (30/489) (11)	16.9% (21/124) (299)
**CDH1**	7.32% (21/287) (296); 0.52% (1/192) (131)	2.3% (11/489) (11)	–
**BLM**	0.4% (9/2561) (300); 1.27% (4/316) (11)	0.6% (3/489) (11)	–
**RBBP8**	1.04% (2/192) (131); 0.32% (1/316) (11); 1.9% (3/158) (48)	0.2% (1/489) (11)	–
**CDK12**	2.9% (9/316) (11); 4% (21/523) (11)	0.4% (2/489) (11)	–
**TP53BP1**	1.27% (4/316) (11); 0.8% (4/523) (78)	1.4% (7/489) (11)	–
**XRCC1**	0.6% (2/316) (11); 0.8% (4/523) (78)	0.4% (2/489) (11)	–
**MAD2L2/REV7**	0.3% (1/316) (11)	0.3% (2/572) (78)	–
**XRCC5/Ku80**	0.2% (1/523) (78)	–	–
**XRCC6/Ku70**	0.3% (1/316) (11); 0.8% (4/523) (78)	0.2% (1/489) (11)	–
**SLFN11**	0.6% (3/523) (78)	0.8% (4/489) (11)	39% (16/41) (209)

“–” no report found, “LOH” loss of heterozygosity.

## 3 Homologous Recombination Deficiency in Breast Cancer

Breast cancer is the most common cancer among women and ranks first and fifth among causes of incidence and mortality, respectively, compared with all other cancers in both sexes in 2020 worldwide. According to the IARC (38), an estimated 2,261,419 (11.72% of all cancer types) new incidences and 684,996 (6.88% of all cancer types) deaths from breast cancer were recorded worldwide in 2020. Similar to ovarian cancer, DNA repair pathways are frequently anomalous in breast cancer, leading to the accumulation of DNA damage and genomic instability. The *BRCA1* and *BRCA2* genes are associated with hereditary breast and ovarian cancer (85). BRCA1 and BRCA2 play a significant role in DNA repair, especially as components of the FANC/BRCA DNA damage response pathway (**Figure 1**). This DNA repair pathway is a highly conserved system involved in the DSB response *via* HR and in the BER pathway for the repair of DNA single-strand breaks (SSBs) (9). The PARP enzyme plays a decisive role in this pathway and is critical for resolving the stalled replication forks. Inhibition of PARP during the base excision process requires BRCA-dependent HRR to resolve it. Targeting HR-related genes (**Table 2**) has potential for destabilizing tumor genomic integrity (13). Clinical trials confirmed that HRD is necessary for the sensitivity to DNA-damaging agents (e.g., cisplatin) and PARPIs (106).

**Table 2:** Prevalence of mutation, LOH, and promoter hypermethylation in breast cancer.

Genes	Mutation [%(proportion)]	LOH [%(proportion)]	Methylation [%(proportion)]
**BRCA1**	5% (15/300) (45); 16.67% (20/120) (86); 7.07% (7/99) (87); 10.6% (409/3,844) (88); 7.35% (201/2733) (89); 9.7% (97/999) (90); 14.7% (118/802) (91)	17.1% (12/70) (92); 44% (16/36) (50); 6.8% (10/147) (93); 41.6% (69/166) (94)	63.9% (46/72) and 27.5% (22/80) (95); 13.33% (8/60) (96)
**BRCA2**	2% (6/300) (45); 5.83% (7/120) (86); 11.11% (11/99) (87); 5.2% (157/3,024) (88); 5.01% (137/2733) (89); 3.5% (35/999) (90); 1.1% (9/802) (91)	14.3% (10/70) (92); 50% (4/20) (50); 31.3% (52/166) (94); 93% (27/29) (97)	69.4% (50/72) and 12.5% (10/80) (95); 15.52% (9/58) (96)
**RAD50**	1.03% (1/97) (98)	32.4% (44/136) (99)	–
**RAD51**	0.2% (2/999) (100)	55% (20/36) (101); 24.41% (31/127 ) (102); 29.4% (40/136) (99)	5.26% (2/38) (96)
**RAD51B**	1.41% (2/142) (65)	47.1% (64/136) (99)	–
**RAD51C**	1.41% (2/142) (65); 5.25% (15/286) (103); 0.53% (23/4309) (104)	0.13% (1/770) (68); 33.8% (46/136) (99)	8% (4/50) (96); 14.46% (23/159) (105); 3.64% (2/55) (106)
**RAD51D**	0.1% (1/894) (107)	36.8% (50/136) (99)	–
**PALB2**	5.7% (404/7093) (108); 5.9% (34/571) (104); 3.4% (33/972) (109); 0.8% (241/30,025) (110); 1.2% (11/937) (111)	2.66% (2/77) (112); 0.72% (7/972) (109); 0.13% (7/770) (68); 14.7% (20/136) (99)	16.7% (8/48) (96); 4.6% (6/130) (76); 95.35% (41/43) (113)
**FANCA**	0.4% (1/255) (44); 0.81% (1.124) (114)	60.3% (82/136) (99)	–
**FANCD2**	1.01% (1/99) (87); 1.2% (3/247) (115); 0.9% (2/216) (116)	22.1% (30/136) (99)	60% (71/118) (117)
**FANCF**	0.5% (4/817) (118); 0.9% (2/216) (116)	17.7% (24/136) (99)	4.04% (4/99) (119); 0.8% (91/120) (120)
**FANCI**	0.4% (1/255) (44); 0.26% (1/392) (121), 2.32% (28/1207) (41); 1.01% (1/99) (87); 5.52% (9/163) (122); 0.81% (1.124) (114)	0.3% (2/816) (118)	–
**FANCM**	0.4% (1/255) (44); 1.5% (6/392) (121); 7.97% (13/163) (122); 0.81% (1.124) (114); 1.4% (6/427) (123); 0.7% (2/286) (124); 1.03% (16/1547) (125)	0.8% (6/770) (68); 35.3% (48/136) (99)	2.33% (1/43) (113); 2.7% (113)
**NBN/NBS1**	1.74% (59/3388) (85); 1.03% (1/97) (126); 0.22% (8/3617) (104); 0.16% (14/8612) (127); 1.2% (53/4566) (128); 1.7% (4/235) (129)	3.33% (1/30) (130); 1.5% (2/136) (99)	8.93% (5/56) (96)
**BARD1**	2% (68/3,388) (85); 0.2% (7/3,667) (104); 0.52% (1/192) (131); 0.18% (52/28,536) (110); 1.67% (2/120) (132); 0.53% (5/937) (111); 0.33% (7/2134) (133)	0.3% (1/330) (134)	10.34% (6/58) (96)
**ATM**	7.1% (5/70) (92); 2.42% (3/124) (114); 1.56% (3/192) (131); 4.6% (55/1207) (41); 1.5% (81/5589) (135); 0.98% (329/33409) (85); 0.94% (274/29229) (110); 0.4% (30/7,657) (136); 5% (3/60) (137)	67% (14/23) (138); 40% (298/745) (138); 19% (13/70) (92); 0.4% (3/770) (68); 48.5% (66/136) (99)	13.79% (8/58) (96); 91.4% (174/190) (139); 53.2% (33/62) (140); 97.4% (223/229) (141); 81% (102/126) (142); 58% (29/51) (143); 25.58% (11/43) (113)

Although *BRCA1* and *BRCA2* have been studied extensively, other genes are also involved in the occurrence of breast cancer. In 3,388 breast cancer patients who underwent genetic testing for 25 genes, nearly half of the pathogenic variants were in the *BRCA1* (24%) and *BRCA2* (24.5%) genes. The remaining 51.5% of pathogenic variants were detected in other genes tested including *CHEK2* (11.7%), *ATM* (9.7%), *PALB2* (9.3%), and Lynch syndrome genes (7%); other genes accounted for the remaining 13.8%. The same study showed that pathogenic mutations in *BRCA1*, *PALB2*, *BARD1*, *BRIP1*, and *RAD51C* are significantly more prevalent than those of other genes in triple negative breast cancer (TNBC) (85). In a study by Lang et al. (144) using NGS based sequencing, the prevalence of somatic *BRCA1* and *2* mutation in sporadic breast cancer cases which carries germline-*BRCA* (g*BRCA*) mutations was 3.5% (15/416). Among these, 1.9% (8/416) and 1.7% (7/416) were *BRCA1* and *2*, respectively. In the same study, somatic *BRCA* mutation in g*BRCA*-negative cases was not detected; indicating somatic *BRCA* mutation in g*BRCA*-negative cases is rare. The prevalence of pathogenic mutations, LOH, and promoter hypermethylation in breast cancer is summarized in **Table 2** based on recently published data.

## 4 Homologous Recombination Deficiency in Colorectal Cancer

CRC is the third most common cancer worldwide. In 2020 alone, the incidence of CRC was estimated at 1,931,590 (10% of all other cancers) worldwide, and it is the second most common cause of cancer-related deaths 935,173 (9.39% of all other cancers) in both sexes after lung cancer (38). Most CRC occurrences are sporadic and are not related to genetic predisposition or family history; however, 20–30% of patients with CRC have a positive family history, and 5% of these tumors arise from genetic predisposition (145). Mutation is a frequent event in CRC. According to TCGA (146), 16% of colorectal carcinomas are hyper-mutated; of these, 75% have high microsatellite instability, typically with hypermethylation and MLH1 silencing, and 25% have somatic mismatch repair gene and polymerase ϵ (POLE) mutations. The prevalence of mutations in *APC*, *TP53*, *SMAD4*, and *PIK3CA* was also reported, as well as *KRAS* mutations. Recurrent copy number alterations (e.g., *ERBB2* amplification), chromosomal translocations, such as the fusion of NAV2 and the WNT pathway member *TCF7L1*, and biallelic inactivation of *APC* were among the common features leading to LOH in CRC. These and other findings confirmed the differences in both oncogenes and tumor suppressor genes involved in CRC compared with those in breast and ovarian cancers. However, a recent study from Japan revealed moderate genomic alterations in DNA repair genes including *BRCA2*, *ATM*, and *NBN* in CRC patients (147). High penetrance genes in HBOC differ from those in CRC; high penetrance genes in CRC are *APC*, *MLH1*, *MSH2*, *MSH2/MUTYH*, *SMAD4*, *MAP*, and *APC/PMS2*; moderate penetrance genes are *MSH6* and *PMS2*; and low penetrance genes are *APC* p.I1307K and *MUTYH* mono-allelic (148).

The methylation profile of CRC differs from that of other cancers; it is characterized by global hypomethylation and promoter-specific DNA hypermethylation. At the whole genome level, CRC has 10–40% lower levels of absolute methylation than normal colonic tissue (149, 150). Global DNA hypomethylation, which is accompanied by genomic instability and tumor initiation, is primarily due to loss of methylation within repetitive elements such as long interspersed nuclear element-1 (*LINE-1*) and Arthrobacter luteus restriction endonucleases (Alu), and it is supposed to contribute to CRC initiation by enhancing genomic instability. On the other hand, genes methylated in CRC are established tumor suppressor genes, and 50% of these are also methylated in the normal colonic epithelium. As a result, methylation in CRC can have three phenotypes according to the CpG-island methylator phenotype (CIMP), i.e., CIMP-high, CIMP-low, and non-CIMP tumors (151). Similar to breast and ovarian cancers, the prevalence of mutation, LOH, and promoter hypermethylation in 35 HR-related genes is described based on recently published data (**Table 3**).

**Table 3:** Prevalence of mutation, LOH, and promoter hypermethylation in colorectal cancer.

Genes	Mutation [%(proportion)]	LOH [%(proportion)]	Methylation [%(proportion)]
**BRCA1**	0.29% (16/5481) (152); 0.28% (3/1058) (153); 0.4% (2/450) (148); 4% (25/619) (154); 3.2% (17/534) (78)	—	—
**BRCA2**	0.34% (5/1474) (152); 0.76% (8/1058) (153); 0.9% (4/450) (148); 6.8% (40/619) (154); 7.1% (38/534) (78)	0.2% (1/592) (78)	10.6% (8/78) (155)
**RAD50**	3.2% (18/619) (154)	0.5% (3/592) (78)	—
**RAD51**	0.5% (3/619) (154)	1.7% (10/592) (78)	—
**RAD51B**	0.8% (5/619) (154)	0.5% (3/592) (78)	—
**RAD51C**	0.08% (1/1260) (156); 0.8% (5/619) (154)	–	—
**RAD51D**	1% (6/619) (154)	0.2% (1/592) (78)	—
**PALB2**	0.44% (3/680) (12); 0.19% (2/1058) (153); 0.4% (2/450) (148); 2.6% (16/619) (154);	0.3 (3/592) (78)	—
**FANCA**	3.1% (19/619) (154)	0.7% (4/592) (78)	—
**FANCD2**	2% (1/50) (157); 4% (25/619) (154)	—	—
**FANCF**	1.3% (8/619 (154)	0.2% (1/592) (78)	—
**FANCI**	2.1% (13/619) (154)	0.2% (1/592) (78)	—
**FANCM**	2% (1/50) (158); 5% (31/619) (154)	2% (1/50) (158)	—
**NBN/NBS1**	0.2% (2/1058) (153); 3.2% (20/619) (154)	—	—
**BARD1**	0.1% (1/1058) (153); 2.33% (1/43) (159); 0.08% (1/1260) (153); 1.1% (7/619) (154)	—	—
**ATM**	0.74% (5/680) (12); 0.95% (10/1058) (153); 0.9% (4/450) (148); 10.3% (64/619) (154)	0.74% (5/680) (12); 2.33% (1/43) (159)	16.67% (13/78) (155)
**ATR**	18.8% (9/48) (160); 4.5% (34/619) (154)	—	—
**MRE11A**	0.3% (3/1006) (161); 2.6% (16/619) (154)	0.2% (1/592) (78)	—
**BRIP1**	0.16% (2/1260) (156); 2.6% (16/619 (154)	—	—
**XRCC1**	1.8% (11/619) (154)	—	—
**CHEK2**	0.23% (1/430) (162); 0.4% (5/1260) (156); 2.65% (4/151) (163); 5.8% (36/619) (154)	0.5% (3/592) (78)	—
**EMSY**	3.2% (20/619) (154);	—	—
**TP53**	0.23% (1/430) (162); 0.1% (1/1058) (153); 0.66% (1/151) (163); 51.7% (320/619) (154)	1.4% (8/292) (78)	—
**STK11**	0.08% (1/1260) (156); 0.8% (5/619) (154)	0.5% (3/292) (78)	—
**PTEN**	60% (87/146) (164); 8.2% (51/619) (154)	23% (6/26) (164)	11.86% (10 (165) (164)
**CDH1**	1.33% (2/151) (163); 2.9% (18/619) (154)	0.2% (1/592) (78)	17.7% (3/17) (166); 87% (53/61) (167)
**CDK12**	5.2% (32/619) (154)	—	—
**BLM**	1.62% (3/185) (168); 1.9% (12/619) (154)	50% (1/2) (168)	—
**TP53BP1**	6.3% (5/124) (169); 6.5% (40/619) (154)	1.5% (9/592) (78)	—
**ERCC1**	0.8% (5/619) (154)	28.3% (43/152) (158)	—
**RBBP8**	1.6% (6/619) (154)	0.2% (1/592) (78)	—
**MAD2L2/REV7**	1% (6/619) (154)	0.5% (3/592) (78)	—
**XRCC5/Ku80**	1.4% (9/619) (154)	—	—
**XRCC6/Ku70**	2.1% (13/619) (154)	—	—
**SLFN11**	2.4% (15/619) (154)	0.2% (1/592) (78)	55.47% (71/128) (170)

“^—^” no report found, “LOH” loss of heterozygosity.

## 5 Homologous Recombination Deficiency in Pancreatic Cancer

Pancreatic cancer (PC) is one of the most devastating types of cancer. PC has a low 5-year survival rate of 9%, and the development of new therapeutics is urgent. The global incidence and mortality of PC in 2020 were 2.6% (495,773 of all cancer types) and 4.68% (466,003 of all cancer types), respectively. PC is the 12th and 6th leading cause of cancer-related incidence and deaths in the world, respectively (38). Although most PC cases occur sporadically, familial (individual having two or more first-degree relatives diagnosed with PC) and hereditary syndrome PC account for 10% of cases. Hereditary cancer syndromes associated with increased risk of PC include Lynch syndrome, familial adenomatous polyposis, Peutz-Jeghers syndrome, atypical multiple mole melanoma, and familial and hereditary breast and ovarian cancer syndrome. Hereditary cancer syndrome accounts for 3% of PC cases, whereas familial PC accounts for 4–10% of cases (171, 172). Familial PC includes individuals with two or more affected first-degree relatives with PC excluding patients with hereditary syndrome. The risk of PC increases with the number of affected first-degree relatives. A study of 838 families including 5,179 individuals showed that the relative PC risk was 4.5 [95% confidence interval (CI): 0.5–16.3) among 1,253 cases with one affected first-degree relative, 6.4 (95% CI: 1.8–16.4) among 634 cases with two affected first-degree relatives, and 32 (95% CI: 10.4–74.7) in 106 cases with three or more affected first-degree relatives (173).

The specific hereditary pancreatic susceptibility genes are *PRSS1*, *SPINK1*, *GGT1*, *CTRC*, and *CFTR*, and mutations in these genes cause early onset PC. However, mutations in these genes are rare and account for a small proportion of PC cases, although the cumulative risk at 70 years age reaches 7–40% with early onset PC (174). Familial PC susceptibility genes include *BRCA2*, *ATM*, *PALB2*, *CDKN2A*, *PRSS1*, *STK11*, *MLH1*, and *MSH2* (175). Recent findings showed the association of PC with genetic alterations in *BRCA2*, *BRCA1*, *ATM*, *CHECK2*, *PALB2*, *FANCC*, and *CDKN2A* genes (176–178). Mutations in *BRCA2* are among the most common genetic mutations involved in familial pancreatic ductal carcinoma. *BRCA* mutation predisposes to PC, and PC more frequently affects *BRCA2* mutation carriers than *BRCA1* carriers. Among 204 *BRCA* mutation carriers with PC, 42.7% (87/204) had *BRCA1* mutations and 57.3% (117/204) had *BRCA2* mutations (179). The prevalence of mutation, LOH, and promoter hypermethylation of 35 HR genes is described according to recently published data in PC (**Table 4**).

**Table 4:** Prevalence of mutation, LOH, and promoter hypermethylation in pancreatic cancer.

Gene	Mutation [%(proportion)]	LOH [%(proportion)]	Methylation [%(proportion)]
**BRCA1**	42.7% (87/204) (179); 2.4% (1/42) (180); 0.3% (1/332) (181); 2.4% (15/615) (177); 0.6% (18/3,030) (178); 1.34% (4/298) (176); 0.4% (3/854) (182); 1.3% (5/456) (183)	20% (10/50) (177); 50% (2/4) (184); 2% (2/100) (185)	8.3% (1/12) (186); 70.6% (12/17) (186); 34.3% (12/35) (186); 60.3% (35/58) (186); 46% (22/48) (187)
**BRCA2**	57.3% (117/204) (179); 26.2% (11/42) (180); 2.11% (7/332) (181); 5.7% (35/615) (177); 1.9% (59/3,030) (178); 1.34% (4/298) (176); 1.41% (12/854) (182); 5.56% (3/54) (188); 0.8% (5/638) (189); 2.1 (8/456) (183)	40% (20/50) (177); 75% (3/4) (184); 6% (6/100) (185)	—
**RAD50**	0.32% (2/615) (177)	3.7% (4/109) (115); 0.32% (2/615) (177); 0.6% (1/183) (78)	—
**RAD51**	—	0.9% (1/109) (115)	—
**RAD51B**	—	1.8% (2/109) (115)	—
**RAD51C**	0.1% (3/3030) (178)	0.1% (3/3030) (178); 0.34% (1/289) (190); 2.8% (3/109) (115)	—
**RAD51D**	0.16% (1/615) (177)		—
**PALB2**	2.4% (1/42) (180); 0.16% (1/615) (177); 0.4% (12/3030) (178); 0.34% (1/298) (176); 0.23% (2/854) (182); 3.7% (2/54) (188); 0.8% (5/638) (191)	0.16% (1/615) (177); 2% (2/100) (185)	—
**FANCA**	0.3% (1/456) (183)	—	—
**FANCD2**	1% (4/456) (183)	3.7% (4/109) (115)	—
**FANCF**	2.8 (3/109) (192)	0.9% (1/109) (115)	—
**FANCI**	—	—	—
**FANCM**	0.47% (3/638) (191); 1.8% (7/456) (183); 1.8% (7/456) (183)	2.8% (3/109) (115)	—
**NBN/NBS1**	0.16% (1/615) (177); 0.13% (4/3030) (178)	0.9% (1/109) (115)	—
**BARD1**	0.16% (1/615) (177); 0.13% (4/3030) (178); 0.34% (1/298) (176)	0.49% (1/615) (177); 0.9% (1/109) (115)	—
**ATM**	0.3% (1/332) (181); 1.8% (11/615) (177); 2.28% (69/3030) (178); 3.36% (10/298) (176); 1.17% (10/854) (182); 3.7% (2/54) (188); 2.98% (19/638) (191); 3.7% (14/456) (183)	72.73% (8/11) (177); 5% (5/100) (185); 4.6% (5/109) (115)	—
**ATR**	0.5% (2/456) (183); 0.9% (1/109) (192)	1.8% (2/109) (115)	—
**MRE11A**	0.07% (2/3030) (178)	0.9% (1/109) (115)	—
**BRIP1**	0.17% (5/3030) (178); 1.04% (3/289) (190)	0.34% (1/289) (190); 2.8% (3/109) (115)	—
**XRCC1**	0.6% (1/179) (78)	—	—
**CHEK2**	2.28% (14/615) (177); 1.09% (33/3030) (178); 1.68% (5/298) (176)	1.95% (12/615) (177); 2.8% (3/109) (115)	—
**EMSY**	0.5% (2/456) (183); 0.9% (1/109) (192)	—	—
**TP53**	89.8% (344/456) (183); 50.5% (55/109) (192); 0.35% (1/289) (190); 0.2% (6/3030) (178)	0.34 (1/289) (190); 5.5% (6/109) (115)	—
**STK11**	0.16% (1/615) (177)	4.6% (5/109) (115)	—
**PTEN**	0.3% (1/456) (183); 0.9% (1/109) (192)	0.6% (1/183) (78); 1.8% (2/109) (115)	–
**CDH1**	0.03% (1/3030) (178); 0.8% (3/456) (183)	—	10.5% (6/57) (186); 50% (1/2) (166); 38% (19/50) (193)
**CDK12**	0.5% (2/456) (183)	—	—
**BLM**	0.49% (3/615) (177)	0.33% (2/615) (177); 1.8% (2/109) (115)	—
**TP53BP1**	0.5% (2/456) (183); 0.9% (1/109) (192)	—	—
**ERCC1**	0.6% (1/179) (78)	0.9% (1/109) (115)	—
**RBBP8**	0.9% (1/109) (192)	2.8% (3/109) (115)	—
**MAD2L2/REV7**	—	1.1% (2/183) (78)	—
**XRCC5/Ku80**	0.6% (1/179) (78)	1.8% (2/109) (115)	—
**XRCC6/Ku70**	0.3% (1/456) (183); 0.6% (1/179) (78)	—	—
**SLFN11**	—	—	

“^—^”no report found, “LOH” loss of heterozygosity.

## 6 Homologous Recombination Deficiency in Non-Small Cell Lung Carcinoma

Lung cancer is the leading cause of cancer-related morbidity and mortality in both genders worldwide. According to the IARC, the estimated number of incidences and deaths from lung cancer was 2,206,771 (11.44%) and 1,796,144 (18.04%) of all cancers worldwide in 2020, respectively (38). The majority of lung cancer cases are associated with smoking or the use of different tobacco products, although other factors such as asbestos, air pollution, radon gas exposure, and chronic infection also contribute to lung carcinogenesis (194). Inherited and acquired mechanisms of lung cancer susceptibility have been proposed, although they are rare. For instance, germline T790M mutation predisposes to a unique hereditary lung cancer syndrome that affects never-smokers and accounts for 31% of the estimated risks for lung cancer in never-smoker carriers (195, 196). Lung cancer is highly invasive, rapidly metastasizing, and broadly categorized into two histological groups, small-cell lung carcinomas (SCLCs) and NSCLCs, which grow and spread differently. NSCLCs account for 87% of cases and can be subdivided into three or four subtypes (adenocarcinoma, squamous cell carcinoma, large cell carcinoma, and undifferentiated NSCLCs), whereas SCLCs account for 12% of lung cancer cases (194).

Generally, lung adenocarcinoma is characterized by recurrent aberrations in multiple key pathways, including activation of RTK/RAS/RAF; activation of PI3K-mTOR; alterations of p53, cell cycle regulators, and the oxidative stress pathway; and mutation of various chromatin and RNA splicing factors. The research network of TCGA demonstrated the activation of oncogenes including *KRAS* (32%), *EGFR* (11%), *MET* (7%), *BRAF* (7%), *MDM2* (8%), *CDK4* (7%), *PIK3C4* (4%), and *CCND1* (4%) and the inactivation of tumor suppressor genes such as *TP53* (46%), *CDKN2A* (43%), *KEAP1* (19%), *STK11* (17%), *NF1* (11%), *ATM* (9%), *RBM10* (9%), *ARID1* (7%), *ARID2* (7%), and *RB1* (7%) in lung cancer (197). Analysis of DNA repair genes associated with squamous cell carcinoma showed a correlation between pathogenic mutations of DNA repair genes and tumor mutation burden. Among DNA repair genes *BRCA1*and *BRCA2* showed the greatest mutation frequency and the tumor burden increased in correlation with the number of affected DNA repair genes (198). A study analyzing mutations in the *BRCA 1* and *2* genes in different cancers showed that *BRCA* mutation is associated with increased incidence of non-breast and ovarian cancers in first- and second-degree relatives of high-risk breast cancer patients. Among 337 *BRCA* mutation carriers, the second highest *BRCA* mutation rate was recorded in lung cancer [8.8% (33/337)] after stomach cancer [13.8% (52/337)] (199). In this review, we displayed the prevalence of mutation, LOH, and promoter hypermethylation of 35 HR genes in NSCLC (**Table 5**).

**Table 5:** Prevalence of mutation, LOH, and promoter hypermethylation in NSCLC.

Genes	Mutation [%(proportion)]	LOH [%(proportion)]	Methylation [%(proportion)]
**BRCA1**	4.5% (8/178) (198); 2.9% (7/240) (200); 4.2% (48/1114) (201)	0.15% (1/655) (68); 0.2% (2/1114) (201)	3.8% (6/158) (202)
**BRCA2**	3.9% (7/178) (198); 3.9% (9/240) (200); 5.2 (60/1114) (201)	0.3% (2/655) (68); 0.36% (4/1114) (201)	—
**RAD50**	1.1% (2/178) (198); 11.11% (2/18) (203); 0.8% (2/240) (200); 1.7% (19/1114) (201)	0.6% (7/1114) (201)	—
**RAD51**	0.56% (1/178) (198); 0.4% (1/240) (200); 0.3% (4/1114) (201)	1.2% (14/1114) (201)	—
**RAD51B**	5.56% (1/18) (203); 0.8% (2/240) (200); 0.8% (9/1114) (201)	0.2% (2/1114) (201)	—
**RAD51C**	0.4% (1/240) (200); 1% (11/1114) (201)	0.09% (1/114) (201)	—
**RAD51D**	0.4% (1/240) (200); 0.6% (7/1114) (201)	0.4% (5/1114) (201)	—
**PALB2**	2.25% (4/178) (198); 1.7% (4/240) (200); 2.3% (26/1114) (201)	0.09% (1/1114) (201)	—
**FANCA**	2.25% (4/178) (198); 11.11% (2/18) (203); 2.5% (6/240) (200); 1.5% (17/1114) (201)	1.1% (12/1114) (201)	—
**FANCD2**	1.2% (14/1114) (201)	0.3% (3/1114) (201)	—
**FANCF**	0.9% (9/1114) (201)	0.15% (1/655) (68); 0.2% (2/1114) (201)	14% (22/126) (202)
**FANCI**	1.8% (19/1114) (201)	—	—
**FANCM**	5.6% (64/1114) (201)	0.5% (6/1114) (201)	—
**NBN/NBS1**	3.75% (17/453) (204); 1.7% (4/240) (200); 1.4% (16/1114) (201)	—	—
**BARD1**	1.1% (2/178) (198); 3.9% (9/240) (200); 1.9% (22/1114) (201)	0.36% (4/1114) (201)	—
**ATM**	4.5% (8/178) (198); 5.56% (1/18) (203); 7.9% (19/240) (200); 7.6% (87/1114) (201); 11.9% (12/101) (205)	0.61% (4/655) (68)	—
**ATR**	5.6% (10/178) (198); 5.56% (1/18) (203); 3.3% (8/240) (200); 4.5% (52/1114) (201)	0.2% (2/1114) (201)	—
**MRE11A**	1.7% (4/240) (200); 1.6% (18/1114) (201)	0.15% (1/655) (68); 0.27% (3/1114) (201)	—
**BRIP1**	4.6% (11/240) (200); 2.5% (28/1114) (201)	0.5% (3/655) (68)	—
**XRCC1**	1% (11/1114) (201)	—	—
**CHEK2**	1.7% (3/178) (198); 1.3% (3/240) (200); 1.9% (22/1114) (201);	0.09 (1/1114) (201)	—
**EMSY**	2.8% (32/1114) (201)	0.2% (2/1114) (201)	—
**TP53**	66.7% (4/6) (206); 20% (46/230) (197); 39.4% (20/1078) (205); 27.8% (5/18) (203); 59.2% (150/240) (200); 67.7% (775/1114) (201)	0.9% (10/1114) (201)	—
**STK11**	7.4% (17/230) (197); 1.8% (20/1078) (205); 27.8% (5/18) (203); 23.3% (56/240) (200); 9.7% (111/1114) (201)	65% (80/124) and 11% (7/62) (207); 0.4% (5/1114) (201)	—
**PTEN**	1.8% (20/1078) (205); 3.3% (8/240) (200); 5.9% (67/1114) (201)	3.1% (36/1114) (201)	—
**CDH1**	1.3% (3/240) (200); 1.8% (20/1114) (201)	0.09% (1/1114) (201)	20% (4/20) (166); 48% (11/23) and 76% (32/42) (208)
**CDK12**	11.11% (2/18) (203); 1.3% (3/240) (200); 3.2% (37/1114) (201)	0.09% (1/1114) (201)	—
**BLM**	2.9% (7/240) (200); 1.8% (20/1114) (201)	—	—
**TP53BP1**	2.9% (7/240) (200); 2.6% (30/1114) (201)	1.4% (16/1114) (201)	—
**ERCC1**	0.2% (2/1114) (201)	—	—
**RBBP8**	1.1% (12/1114) (201)	—	—
**MAD2L2/REV7**	0.3% (3/1114) (201)	0.27% (3/1114) (201)	—
**XRCC5/Ku80**	1.6% (18/1114) (201)	0.36% (4/1114) (201)	—
**XRCC6/Ku70**	1.3% (15/1114) (201)	0.09% (1/1114) (201)	—
**SLFN11**	2.3% (26/1114) (201)	0.3% (3/1114) (201)	13.6% (3/22) (209)

“—” no report found, “LOH” loss of heterozygosity

## 7 Homologous Recombination Deficiency in Prostate Cancer

Prostate cancer is the most prevalent cancer in men next to lung cancer, according to the IARC in 2020 (38), it ranked 2^nd^ and 5^th^ in its incidence and mortality among men, respectively. The estimated number of incidences and deaths from prostate cancer in 2020 were 1,414,259 (14.05%) and 375,304 (6.79%) worldwide, respectively. Prostate cancer is characterized by a high degree of heritability, and genetic components contribute significantly to disease incidence (210). A large cohort study conducted in the Nordic region that analyzed the different cancer heritability risks in monozygotic and dizygotic twins identified a risk of prostate cancer of 57% (95% CI: 51–63), which was higher than that of other cancer types such as ovarian cancer at 39% (95% CI: 23–55) and breast cancer at 31% (95% CI: 11–51) (210). Although the high rate of heritability of prostate cancer has been demonstrated in patients with a positive family history, candidate genes that contribute to prostate cancer heritability have not been identified except *HOXB13* (211). Recurrent mutation of the *HOXB13* gene at G84E was identified in many families. The *HOXB13* G84E allele accounts for approximately 5% of hereditary prostate cancer (211). A study including 2,443 prostate cancer families of European descent detected at least one *HOXB13* G84E mutation carrier, among 112 prostate cancer families (4.6%) (212). Moreover, a study conducted in unrelated subjects of European descent revealed, *HOXB13* G84E mutation was detected in 1.4% (72/5083) and 0.07% (1/1401) of participants with- and without prostate cancer, respectively (P<0.05) (213). Another study comprising 9,012 men diagnosed with different cancers showed a rate of 0.54% (49/9012) of *HOXB13* G84E mutation carriers, of whom 1.4% (19/1362) were positive for prostate cancer compared with 0.4%(23/5,898) of *HOXB13* G84E mutation carriers without prostate cancer (*p* < 0.05) (214). Prostate cancer has a genetic origin in <5% of cases, and this risk becomes higher when high penetrance genes such as *HOXB13* are involved (215). Recent gene linkage studies identified additional prostate cancer susceptibility genes such as *HPC1*, *HPC2/ELAC2*, *MSR1*, *BRCA1*, *BRCA2*, and *BRIP1* (216).

An estimated 20% of patients with prostate cancer have a positive family history, which can be attributed not only to shared genes, but also to a shared pattern of exposure to environmental carcinogens and common lifestyle habits (216). Additional challenges in the management of prostate cancer include its genetic heterogeneity and a high rate of sporadic cases; many common genetic variants are associated with prostate cancer, explaining the familial clustering of the disease rather than hereditary causes (211). The importance of both germline and somatic alterations in DNA repair genes is suggested by the fact that carriers of mutations in these genes are at a high risk of developing aggressive or metastatic prostate cancer (211). Deleterious mutations of *BRCA1* and *BRCA2* are associated with increased risk of prostate cancer and experienced very aggressive course of the disease (215). However, studies focusing on families with only prostate cancer failed to identify a significant number of *BRCA1* or *BRCA2* mutations, indicating their minimal role in hereditary prostate cancer predisposition (211). A comprehensive genomic analysis of 1,013 prostate cancer patients revealed the indispensable role of alterations in DNA repair genes (78). Here, the prevalence of mutation, LOH, and promoter hypermethylation in 35 DNA repair genes in prostate cancer was described based on recently published data (**Table 6**).

**Table 6:** Prevalence of mutation, LOH, and promoter hypermethylation in prostate cancer.

Genes	Mutation [%(proportion)]	LOH [%(proportion)]	Methylation [%(proportion)]
**BRCA1**	0.6% (6/1013) (38); 0.3% (3/494) (78); 1.2% (4/333) (217); 1% (5/504) (218); 1.8% (8/444) (219)	1.3% (13/1013) (38); 1.2% (6/489) (78);	25-75% (220)
**BRCA2**	2.9% (6/1013) (38); 1.6% (8/494) (78); 2.7% (9/333) (217); 5.2% (26/504) (218); 8.3% (37/444) (219)	2.5% (25/1013) (38); 3.5% (17/489) (78); 0.6% (2/333) (217); 3% (15/501) (218); 2.9% (13/444) (219)	—
**RAD50**	0.4% (4/1013) (38); 0.2% (1/494) (78); 0.6% (3/504) (218); 0.7% (3/444) (219)	1.2% (12/1013) (38); 0.8% (4/489) (78); 1.2% (4/333) (217); 0.4% (2/501) (218); 1.1% (5/444) (219)	—
**RAD51**	0.1% (1/1013) (38); 0.2% (1/494) (78);	0.8% (4/489) (78); 1.8% (6/333) (217); 0.4% (2/501) (218); 1.4% (6/444) (219)	—
**RAD51B**	0.6% (3/494) (78)	0.6% (3/489) (78); 1.2% (4/333) (217); 0.4% (2/501) (218); 1.1% (5/444) (219)	—
**RAD51C**	0.2% (1/504) (218)	1.3% (13/1013) (38); 0.6% (3/489) (78); 0.3% (1/333) (217); 0.5% (2/444) (219)	—
**RAD51D**	0.3% (1/333) (217); 0.2% (1/504) (218); 0.7% (3/444) (219)	0.6% (6/1013) (38); 0.6% (3/489) (78); 1.2% (4/333) (217); 0.2% (1/444) (219)	—
**PALB2**	1% (10/1013) (38); 0.6% (3/494) (78); 0.3% (1/333) (217); 1.2% (6/504) (218); 1.4% (6/444) (219)	0.3% (1/333) (217); 0.6% (3/501) (218); 0.2% (1/444) (219)	—
**FANCA**	0.3% (3/1013) (38); 0.3% (1/333) (217); 0.6% (3/504) (218); 0.9% (4/444) (219)	2% (20/1013) (38); 4.7% (23/489) (78); 7.8% (26/333) (217); 2.4% (12/501) (218); 0.2% (1/444) (219)	—
**FANCD2**	0.3% (3/1013) (38); 0.4% (2/494) (78); 0.3% (1/333) (217); 0.7% (3/444) (219)	1.5% (15/1013) (38); 2% (10/489) (78); 0.6% (2/333) (217); 1.1% (5/444) (219)	—
**FANCF**	0.2% (2/1013) (38); 0.2% (1/494) (78); 0.3% (1/333) (217); 0.5% (2/444) (219)	0.5% (2/444) (219)	—
**FANCI**	0.3% (3/1013) (38); 0.4% (2/494) (78); 0.3% (1/333) (217);	0.7% (3/444) (219)	—
**FANCM**	0.6% (6/1013) (38); 0.4% (2/494) (78); 0.3% (1/333) (217); 1.6% (7/444) (219)	0.2% (489) (78)	—
**NBN/NBS1**	0.6% (6/1013) (38); 0.6%(3/494) (78); 0.3% (1/333) (217); 0.4% (2/504) (218); 0.7% (3/444) (219)	0.2% (2/1013) (38); 0.4% (2/489) (78); 0.2% (1/444) (219)	—
**BARD1**	0.6% (6/1013) (38); 0.8% (4/494) (78); 0.6% (3/504) (218); 0.9% (4/444) (219)	0.5% (5/1013) (38); 0.2% (1/489) (78); 0.3% (1/333) (217); 0.2% (1/501) (218); 0.9% (4/444) (219)	—
**ATM**	3.8% (38/1013) (38); 4.3% (21/494) (78); 3.9% (13/333) (217); 3.6% (18/504) (218); 6.1% (27/444) (219)	0.8% (8/1013) (38); 1.2% (6/489) (78); 2.1% (7/333) (217); 1.2% (6/501) (218); 1.6% (7/444) (219)	—
**ATR**	1% (10/1013) (38); 1% (5/494) (78); 1.4% (7/504) (218); 1.4% (7/444) (219)	0.5% (5/1013) (38); 0.4% (2/489) (78); 0.3% (1/333) (217); 0.2% (1/444) (219)	—
**MRE11A**	0.5% (5/1013) (38); 0.6% (3/504) (218); 0.9% (4/444) (219)	0.7% (7/1013) (38); 0.4% (2/489) (78); 0.6% (2/333) (217); 0.2% (1/501) (218)	—
**BRIP1**	0.6% (6/1013) (38); 0.6% (3/494) (78); 0.4% (2/504) (218); 0.9% (4/444) (219)	–	—
**ERCC1**	0.3% (3/1013) (38); 0.2% (1/494) (78); 0.5% (2/444) (219)	0.2% (1/489) (78); 0.3% (1/333) (217); 0.7% (3/444) (219)	—
**CHEK2**	0.4% (4/1013) (38); 0.4% (2/504) (218); 1.4% (6/444) (219)	1.2% (12/1013) (38); 1.4% (7/489) (78); 3% (10/333) (217); 0.4% (2/501) (218); 1.4% (6/444) (219)	—
**EMSY**	0.8% (8/1013) (38); 0.4% (2/494) (78); 0.3% (1/333) (217); 1.1% (5/444) (219)	0.4% (4/1013) (38); 0.2% (1.444) (219)	—
**TP53**	18.7% (189/1013) (38); 12.3% (61/494) (78); 6.9% (23/333) (217); 33.5% (169/504) (218); 36.7% (163/444) (219)	2% (20/1013) (38); 4.3% (21/489) (78); 0.6% (2/333) (217); 1.8% (9/501) (218); 3.4% (15/444) (219)	—
**STK11**	0.2% (2/1013) (38); 0.2% (1/494) (78); 0.4% (2/504) (218); 0.2% (1/444) (219)	3.4% (34/1013) (38); 0.2% (1/501) (218); 2.9% (13/444) (219)	—
**PTEN**	4.3% (44/1013) (38); 5.5% (27/494) (78); 2.7% (9/333) (217); 6% (30/504) (218); 6.3% (28/444) (219)	12.2% (124/1013) (38); 17.4% (85/489) (78); 15%(50/333) (217); 12.4% (62/501) (218); 25.7% (114/444) (219)	7.8% (221)
**CDH1**	0.9% (9/1013) (38); 0.8% (4/494) (78); 0.6% (2/333) (217); 1.2% (6/504) (218); 0.9% (4/444) (219)	1.7% (17/1013) (38); 2.9% (14/489) (78); 4.5% (15/333) (217); 0.4% (2/501) (218); 2% (9/444) (219)	69% (70/101) (222); 61% (49/81) (223); 27% (27/101) (224)
**BLM**	0.2% (2/1013) (38); 0.4% (2/504) (218); 0.5% (2/444) (219)	0.1% (1/1013) (38); 0.7% (3/444) (219)	—
**RBBP8**	0.2% (2/1013) (38); 0.2% (1/444) (219)	–	—
**CDK12**	3.3% (33/1013) (38); 2.2% (12/494) (78); 1.8% (6/333) (217); 5.6% (28/504) (218); 5.9% (26/444) (219)	0.7% (7/1013) (38); 0.4% (2/489) (78); 0.6% (2/333) (217); 0.4% (2/501) (218); 0.5% (2/444) (219)	—
**TP53BP1**	0.9% (9/1013) (38); 1.4% (7/494) (78); 0.9% (3/333) (217); 0.5% (2/444) (219)	1.7% (17/1013) (38); 0.8% (4/489) (78); 1.8% (6/333) (217); 1.4% (6/444) (219)	—
**XRCC1**	0.2% (2/1013) (38); 0.3% (1/333) (217); 0.5% (2/444) (219)	0.8% (4/489) (78); 1.5% (5/333) (217); 1.4% (6/444) (219)	–
**MAD2L2/REV7**	0.1% (1/1013) (38); 0.2% (1/494) (78);	0.6% (6/1013) (38); 0.2% (1/489) (78); 0.3% (1/333) (217); 0.5% (2/444) (219)	—
**XRCC5/Ku80**	0.2% (2/1013) (38); 0.2% (1/494) (78); 0.3% (1/333) (217); 0.2% (1/444) (219)	0.5% (5/1013) (38); 0.4% (2.489) (78); 1.2% (4/333) (217); 0.2% (1/444) (219)	—
**XRCC6/Ku70**	0.5% (5/1013) (38); 0.6% (4/494) (78); 0.3% (1/333) (217); 0.2%(1/444) (219)	0.2% (1/489) (78); 0.6% (2/333) (217); 0.7% (3/444) (219)	—
**SLFN11**	0.3% (3/1013) (38); 0.2% (1/494) (78); 0.6% (2/333) (217); 0.2% (1/444)	0.4% (2/489) (78); 0.9% (3/333) (217); 0.2% (1/444) (219)	—

“—” no report found, “LOH” loss of heterozygosity.

## 8 Poly (ADP-Ribose) Polymerase Inhibitor Resistance Mechanism

### 8.1 The Role of PARP in DNA Repair

BER, HR, NHEJ, and micro homology-mediated end-joining (MMEJ) repair SSBs and DSBs. PARP1 is involved in all DNA repair mechanisms. SSBs are primarily repaired by BER (high fidelity DNA repair) using PARP1. DSBs are repaired by three mechanisms: HR, NHEJ, and MMEJ. HRR (high fidelity DNA repair mechanism) of DSBs is performed by recruiting BRCA1 and 2, RAD51, the MRN complex, and ATM, here PARP1 contributes to HR by recruiting MRE11 and NBS1 or by ribosylating BRCA. NHEJ (error-prone DNA repair mechanism) repair of DSBs involves the recruitment of Ku70, Ku80, and DNA-dependent protein kinase catalytic subunit (PKcs); PARP1 prevents the binding of Ku proteins to free DNA ends (first step of NHEJ) and thus inhibits NHEJ. MMEJ (error-prone DNA repair mechanism) repairs DSBs by recruiting Flap Structure-Specific Endonuclease-1 and NBN; PARP1 prevents binding of Ku proteins and directs DSBs to an alternate end-joining (MMEJ) repair pathway (225–227).

### 8.2 Mechanisms of PARP Inhibition

In the presence of SSBs, PARP1 binds to the SSB site and undergoes poly (ADP- ribosyl) ation, an important step for PARP1 activation; then, the poly(ADP-ribosyl)ated PARP1 recruits the DNA repair complexes BARD1-BRCA1 and MRN, which restore the integrity of DNA through a high fidelity DNA repair mechanism, resulting in cell survival. Inhibition of PARP by PARPIs drive to change the repair mechanism from SSBs to DSBs. PARPIs bind to PARP1 and inhibit its poly (ADP-ribosyl) ation as well as inhibiting BER. In addition, PARPIs prevent the release of PARP from the polymer form, thereby inhibiting the recruitment and binding of DNA damage repair proteins (PARP trapping), which further aggravates the inhibition of BER. Once BER is inhibited by PARPI, SSBs are converted into DSBs, forcing cells to opt for HRR. However, HR can only be used if cells are HR-proficient. HR defects (mutation, LOH, and hypermethylation) in HR-related genes such as *BRCA1* and *2*, *RAD51* and its paralogs (*RAD51B*, *RAD51C*, and *RAD51D*), *FA* genes, *PALB2*, the MRN complex, *BARD1*, *ATM*, *ATR*, and *BRIP1* cause cells to become HR-deficient and unable to repair DSBs. This causes the persistence of DNA DSBs, which leads to genomic instability and cell death. Cells need to activate alternative DNA repair mechanisms such as NHEJ and MMEJ, the only remaining repair mechanisms. Thus, cells are forced to use the two error-prone DSB repair mechanisms, which results in genomic instability and cell death (225–227). PARP1 and PARP2 are constitutively expressed enzymes that peak during the S-phase of the cell cycle and are activated by binding to DNA damage sites. PARPIs are particularly effective in the treatment of high grade ovarian and breast cancers with HR defects, which are characterized by frequent replication of tumor cells, and PARP expression and DNA damage recognition are highest during S-phase. However, the use of PARPIs is not limited to these two cancers. The concept of synthetic lethality or the BRCAness phenotype is wider, and most cancers with HR repair defects benefit from PARPI treatment including pancreatic and prostate cancers (227).

### 8.3 Mechanisms of Resistance to PARP Inhibitors

**Figure 2:**
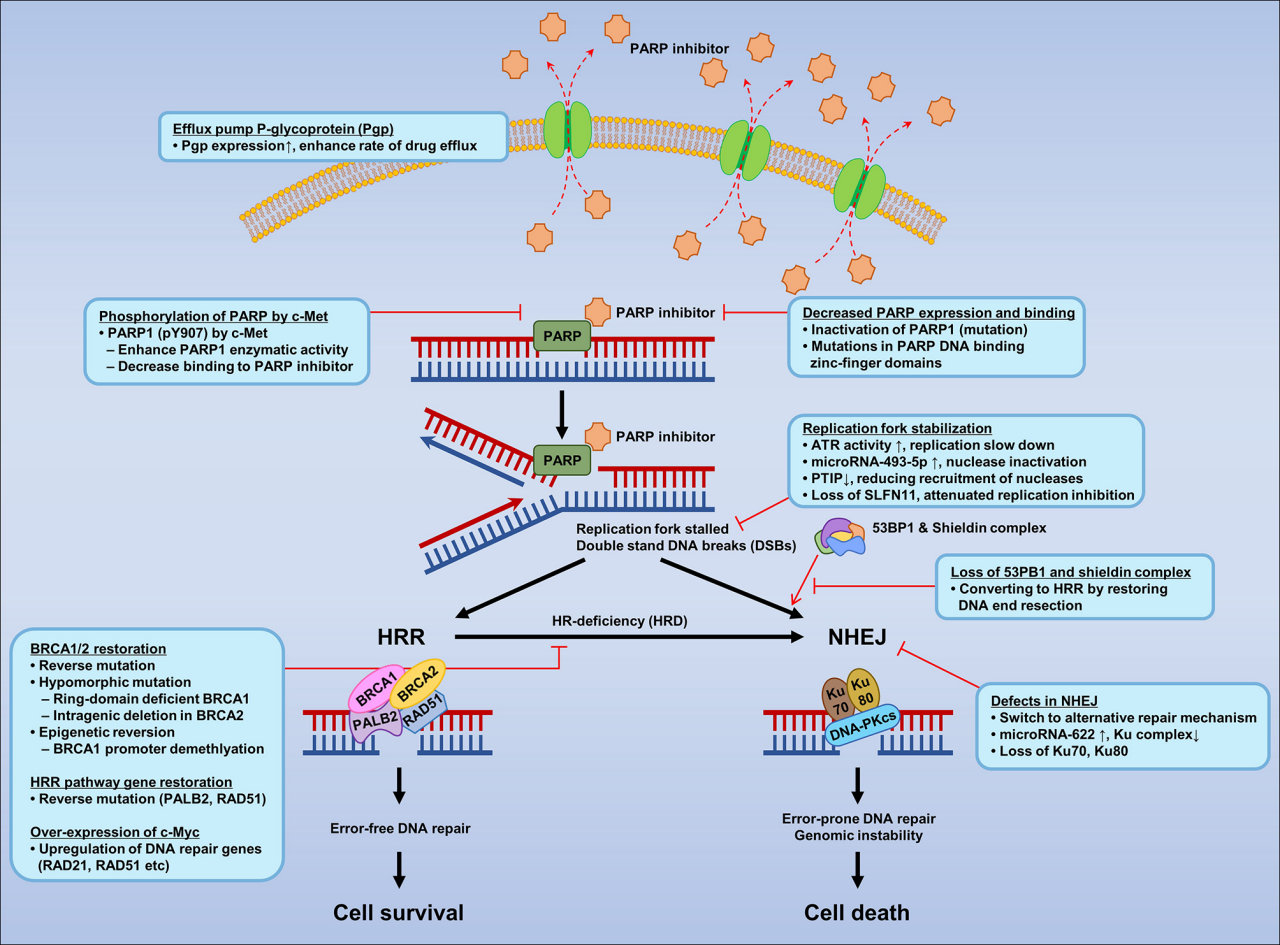
Mechanisms of resistance to PARP inhibitors according to mechanisms of PARPI in DNA repair.

Despite the introduction of new drugs, the emergence of resistance to PARPIs remains a limiting factor (227, 228). Several mechanisms of PARPI resistance have been identified, including restoration of HRR proficiency, switching to alternate repair mechanisms such as NHEJ, replication fork stabilization, drug efflux, decreased PARP expression and binding, secondary mutations in HR-related genes and *RAD51*, regulation by microRNAs, phosphorylation of PARP by c-MET, loss of end resection regulation by 53BP1, epigenetic reversion of methylated promoters, and mutations in the shielding complex among others (**Figure 2**).

#### 8.3.1 Restoration of Homologous Recombination Proficiency

HR proficiency can be restored directly by reverse mutation of *BRCA1* and *BRCA2* mutants (229). Reverse mutation might be elicited by genomic instability due to *BRCA* loss. In addition, the presence of hypomorphic (partial loss of gene function) *BRCA1* mutation leads to selection of cells with restored BRCA function, which confer resistance to PARPIs (230). In a recent study of high-grade ovarian carcinoma, *BRCA* reversion mutation was identified in 18% (2/11) and 13% (5/38) of pretreatment cell-free DNA extracts from platinum refractory and resistant cancers, respectively, compared with 2% (1/48) of platinum-sensitive cancers (*p* < 0.05) (231). Patients without *BRCA* reversion mutation detected in pretreatment circulation cell-free DNA extracts had significantly (*p* < 0.05) longer progression free survival than those with reversion mutation after treatment with rucaparib (9 versus 1.8 months), which decreased the clinical benefit from rucaparib (231). Although genetic reversion of *BRCA1* and *BRCA2* is one of the underlying mechanisms, it does not explain PARPI resistance in all cases. For example, loss of *REV7* (*MAD2L2*) re-establishes CTIP-dependent end resection of DSBs in *BRCA1*-deficient cells, leading to HR restoration and PARPI resistance (232).

Secondary somatic mutations that restore *BRCA1/2* in carcinomas from women with germline *BRCA1/2* mutations predict the resistance to platinum and PARPIs. In cohorts of 64 primary and 46 recurrent ovarian carcinoma patients, secondary somatic mutation was detected in 3.1% (2/64) of primary carcinomas and in 28.3% (13/46) of secondary carcinomas (*p* < 0.05) due to secondary mutations in *BRCA1/2*. In the same study, 46.2% (12/26) of the platinum resistance recurrence cases had secondary mutations that restored BRCA1/2 function compared with 5.3% (1/19) of platinum-sensitive recurrence cases (*p* < 0.05) (165). Furthermore, the reversion mutations were not only detected in *BRCA1/2*, but also in other HRR pathway genes including *RAD51C*, *RAD51D*, and *PALB2* in ovarian, prostate, and breast carcinomas as a mechanism of acquired resistance to platinum-based chemotherapies and PARPIs. Therefore, primary mutations of HR genes cause sensitivity to platinum and PARPI therapy, whereas secondary mutations cause resistance (231, 233). *BRCA2* reversion mutations confer resistance to olaparib and talazoparib in prostate cancer patients. Analysis of circulating cell-free DNA provides information on reversion mutation heterogeneity that is not distinct from that of single solid tumor biopsy, as well as potential indications for monitoring the emergence of PARPI resistance (234).

#### 8.3.2 RING Domain-Deficient BRCA1 and Intragenic Deletion in BRCA2

High expression levels of RING domain-deficient BRCA1 proteins promote cisplatin and PARPI resistance by reducing the DNA repair capacity of BRCA1 in breast cancer cell lines. The BRCA1 185delAG hypomorphic allele, a common inherited mutation located close to the protein translation start site, produces a shortened and nonfunctional peptide. In contrast to the full length BRCA1, the translation start site for the RING domain-deficient BRCA1 protein is located downstream of the frameshift mutation at the BRCA1-Met-297 codon and does not require interaction with BARD1 for stability unlike the full length BRCA1. Functionally, the RING domain-deficient BRCA1 supports RAD51 foci formation, which increases HRR and confers partial PARPI and cisplatin resistance (235). A recent case control association study and functional analysis of BRCA2 identified a hypomorphic missense variant (Y3035S) associated with a moderate risk of breast cancer. However, the role of this hypomorphic missense variant in the resistance to PARPIs and cisplatin remains to be investigated (236). Another study identified new BRCA2 isoforms that are expressed in resistant cell lines as a result of intragenic deletion of the c.6174delT mutation and restoration of the open reading frame. Reconstitution of BRCA2-deficient cells with these reverting BRCA2 alleles made resistant to PARPI and HR proficient (237). This finding indicates that resistance to PARPIs can arise by intragenic deletion mutations in BRCA2.

#### 8.3.3 Epigenetic Reversion of Methylated Promoters

Epigenetic silencing of the promoter region of tumor suppressor genes is one of the mechanisms underlying HRD. For instance, *BRCA1* promoter methylation is an important somatic driver in high grade serious ovarian carcinoma (238, 239). A patient with *BRCA1* promoter methylation who was initially sensitive to PARPIs became resistant after loss of *BRCA1* promoter methylation in the relapsed sample, and the gene was expressed at comparable levels to those in HR-proficient tumors (238). In the same study, analysis of the global methylation status of the primary and reverted samples revealed loss of *BRCA1* promoter methylation. Another study showed *BRCA1* promoter demethylation in therapy-resistant patients and patient-derived xenograft (PDX) tumors. Among 103 TNBC patients, 26 (25.24%) showed *BRCA1* promoter methylation before treatment. Of these, 17 showed pathologic complete response and nine showed partial/no response; the three partial responders underwent post-treatment surgery. Post-treatment *BRCA1* promoter methylation was 2.66-fold lower than that in pretreatment samples, and the mRNA expression of *BRCA1* increased by 12–28-fold. Loss of *BRCA1* promoter methylation was observed in 69.6% (16/23) of therapy-resistant PDX tumors with BRCA1 re-expression (240).

#### 8.3.4 Switch to Alternate Repair Mechanisms

As discussed earlier, DNA DSBs in homologous recombination-deficient cancer cells can be repaired by alternative DNA repair mechanisms such as NHEJ and MMEJ. Thus, the shift from the canonical DNA repair mechanism to alternate repair mechanisms can affect the therapeutic efficacy of PARPIs (228). NHEJ functions throughout the cell cycle, and defects in NHEJ contribute to genomic instability and are associated with the development of chemo-resistance. NHEJ is crucial for determining the sensitivity to PARPIs, as confirmed recently (241). For example, ovarian cancer cells with a 40% deficiency in the NHEJ DSB repair pathway are resistant to PARP inhibition irrespective of HR status. Only NHEJ-competent and HRD cells are sensitive to the PARPI rucaparib, confirming the resistance observed in HRD tumors (241). Therefore defects in NHEJ, the lack of error-prone repair results in resistance to PARPIs. The role of NHEJ in PARPI resistance is related to the error proneness of NHEJ. The errors in repair cause lethal defects in DNA, and the absence of HR results in apoptosis, which is required for PARPI sensitivity (242).

#### 8.3.5 Replication Fork Stabilization

HR-deficient cells are susceptible to replication fork degradation and are sensitive to PARPIs. However, cancer cells possess a mechanism to protect against replication fork degradation known as fork stabilization. Replication fork stabilization is a compensatory mechanism that protects the replication fork, which results in PARPI resistance in the absence of HR competency (105). Another mechanism to stabilize the replication fork is ATR activation in response to SSBs. In this mechanism, CHK1 is phosphorylated by ATR, and activated CHK1 phosphorylates WEE1 and inactivates the sCDC25A and CDC25C phosphatases. Activated WEE1 activates CDK1 and CDK2 to promote G1/S and G2/M cell cycle arrest (243, 244). Another mechanism of replication fork protection was identified by Meghani et al. (245). In this mechanism, miR-493-5p overexpression protects the replication fork from nuclease degradation, subsequently inducing PARPI and platinum resistance in *BRCA2*-mutated carcinomas. In addition, Pax2 transactivation domain-interacting protein (PTIP), which forms nuclear foci for DSBs, can destabilize the MRE11 nuclease installed replication forks. By contrast, loss of PTIP stabilizes nascent DNA strands by blocking degradation in *BRCA1/2* deficient cells, a mechanism that rescues the stalled replication fork and causes PARPI and cisplatin resistance (246).

#### 8.3.6 Decreased PARP Expression and Binding

Deletion of PARP1 using CRISPR/Cas9 gene editing tools in two ovarian cancer cells (one with *BRCA1* mutation and one with *BRCA1* promoter methylation) shows >90% reduction of PARP1 expression in *BRCA1* mutant and promoter methylated cells as measured by immunofluorescence and western blot analysis. Therefore, loss of PARP1 by different mechanisms (e.g., mutation) results in resistance to PARPIs (247). Although *BRCA1* mutant and promoter methylated ovarian cancer cells are synthetically lethal with PARPI, the loss of the target (PARP) results in PARPI resistance. Point mutations that interfere with the PARP1 DNA binding zinc-finger domains cause PARPI resistance and affect PARP1 trapping. PARP1 p.R591C mutation (c.1771C>T) was detected in ovarian cancer patient who showed resistant to olaparib (248). Other mutations that occur outside of the zinc-finger domain of PARP1 also reduce PARP trapping.

#### 8.3.7 Efflux Pump P-Glycoprotein

The multidrug efflux pump P-glycoprotein (Pgp) contributes markedly to chemotherapy resistance by increasing rate of drug efflux. Long-term administration of PARPIs causes selective pressure-induced PARPI resistance mediated by the upregulation of a gene-encoding efflux pump. Pgp recognizes and transports a variety of chemical substrates with hydrophobic in nature (249). The expression of abcb1a and abcb1b, encoding murine Pgp were increased by 2 to 15 fold in 73.3% (11/15) of mice treated with AZD2281 (currently, Olaparib) (249). Upregulation of Pgp expression is considered a mechanism of resistance in *BRCA1* and *BRCA2* mutant cancers treated with PARPIs (250). Upregulation of multidrug resistance gene 1 increases the expression of Pgp and the rate of drug efflux, which decreases the therapeutic effect of PARPIs (251).

#### 8.3.8 Loss of End Resection Regulation by 53BP1 and Mutation in the Shieldin Complex

The 53BP1 is an important regulator of the cellular response to DSBs, and it suppresses HR by stimulating NHEJ of the distal DNA end. Deletion of 53BP1 converts processing of damaged DNA ends into recombination of single-stranded DNA competent for HR. Loss of 53BP1 partially restores the error-free HR and reduces the sensitivity of BRCA1 mutant tumors to PARPIs (7, 252). 53BP1 is crucial for the control of DSB repair, as its presence promotes NHEJ and its loss promotes HR. Inhibiting 5′ end resection is necessary for HRD, and 53BP1 uses Rif1 to impair 5′ end resection. Rif1 inhibits end resection by recruiting CtIP, BLM, and Exo1, which restricts buildup of BRCA1/BARD1 complexes at sites of DNA damage. These mechanisms underlie the effect of 53BP1 on inducing chromosomal aberrations in *BRCA1*-deficient cells. Therefore, loss of 53BP1 favors HR and thus leads to PARPI resistance (253, 254).

The 53BP1 effector complex (shieldin) includes SHLD1, SHLD2, SHLD3, and REV7. Shieldin functions as a downstream effector of 53BP1-RIF1 in preventive DNA DSB repair, whereas deletion of the shieldin complex confers resistance to PARPIs in *BRCA1*-deficient cells. Binding of single-stranded DNA by SHLD2 is critical for shieldin function (255). *BRCA1* mutant cancers show minimal resection of DSBs, which renders them deficient in homology-directed repair and sensitive to inhibitors of PARP1. In *BRCA1* mutants, the resection of DSBs is inhibited by 53BP1, RIF1, and the shieldin complex, and loss/deletion of these factors reduces sensitivity to PARP1 inhibitors (256). Mutations in genes that encode shieldin subunits also cause resistance to PARPIs in *BRCA1*-deficient cells and tumors, resulting in restoration of HR (257). Silencing of shieldin components increases end resection, as extreme resection would make DNA ends unsuitable for repair by NHEJ; this may explain the defective NHEJ in shieldin-depleted cells. Downregulation of 53BP1, as well as that of REV7, confers resistance to PARPIs in *BRCA1* mutant cells. An experiment in *BRCA1*-deficient and shieldin complex knockout cells confirmed the important role of the shieldin complex in controlling PARPI (olaparib) sensitivity. *BRCA1*-depleted cells are highly sensitive to olaparib; however, simultaneous depletion of shieldin components rescues cell viability similar to the effects of depletion of BRCA1 and 53BP1 (258). Therefore, loss of 53BP1, RIF1, and shieldin components is sufficient to bypass the HR function of BRCA1 and confer PARPI resistance.

#### 8.3.9 Overexpression of MicroRNAs

Overexpression of miR-622 is implicated in the development of resistance to PARPIs and cisplatin by restoring HR and impairing NHEJ in *BRCA1*-deficient ovarian cancer. miR-622 suppresses NHEJ by downregulating the Ku complex, thus promoting HR-mediated repair of DNA DSBs in the S-phase of the cell cycle. In addition, overexpression of miRNA-622 in HGSOC patients is correlated with worse survival after platinum chemotherapy, associating miRNA-mediated resistance by rescuing HRR (259). Overexpression of miR-493-5p induces resistance to platinum and PARPIs in *BRCA2* mutant patient-derived cells by targeting DNA repair pathways involved in genomic stability. MiR-493-5p induces resistance by downregulating R-loop processing genes, which increases the R-loop and decreases the single-strand repair pathway, and by downregulating nucleases, which protects the replication fork. HRR is not restored in relation to miR-493-5p mediated cisplatin and PARPI resistance. Overexpression of miR-493-5p is negatively correlated with disease-free survival, especially in *BRCA2* mutant patients and specifically in platinum resistant or refractory disease (245).

#### 8.3.10 Phosphorylation of PARP by C-Met

Phosphorylation of PARP1 at Tyr907 by the receptor tyrosine kinase c-Met causes PARPI resistance. The phosphorylation of PARP1 by c-Met (pY907) enhances PARP1 enzymatic activity and decreases binding to PARPI, resulting in resistance of cancer cells to PARPIs. PARPIs and c-Met inhibitors act synergistically in suppressing the growth of breast and lung cancer cells *in vitro* and in a xenograft model. Detection of pY907 is an indicator of PARPI resistance in combination with a poor response to PARPIs and high c-Met expression (260). PARPIs are commonly used for the treatment of ovarian and breast cancers. A recent study investigating the therapeutic efficacy of PARPIs against hepatocellular carcinoma (HCC) showed discouraging results. The mechanisms underlying the poor efficacy of PARPIs in HCC involve the formation of EGFR and MET heterodimer that interacts with and phosphorylates Y907 of PARP1 in the nucleus, which contributes to PARPI resistance. However, inhibition of both c-Met and EGFR sensitizes HCC cells to PARPIs, although both EGFR and c-Met are usually overexpressed in HCC (261). The use of c-Met and EGFR inhibitors in combination with PARPIs is a potential strategy for the treatment of HCC.

#### 8.3.11 Overexpression of C-Myc

Overexpression of c-Myc increases cisplatin and PARPI resistance by reducing the production of the c-Myc inhibitor BIN1 (bridging integrator 1) which restores the intrinsic PARP-1 activity. Suppression of BIN1 releases the automodification domain of PARP1, which increases its intrinsic catalytic activity for DNA repair, thereby increasing resistance to PARPIs and cisplatin. Conversely, inhibition of c-Myc increases BIN1 abundance, which decreases PARP1 activity and reverses cisplatin and PARPI resistance (262). *Myc* amplification is accompanied by the upregulation of several DNA repair genes, including *RAD21*, *RAD54L*, and *RAD51*, in both breast and ovarian cancer. *RAD51* is the third most significant DNA repair gene associated with Myc expression in TNBC tumor samples. c-Myc regulates PARPI resistance by upregulating RAD51 paralogs, which are important in HRR of damaged DNA. A recent study using TNBC cell lines confirmed that PARPI-resistant cells have increased RAD51 foci, whereas PARPI-sensitive cells show impaired RAD51 foci independent of *BRCA* mutation status. In the same study, pharmacological inhibition of c-Myc by dinaciclib reversed the resistance to PARPIs, confirming the induction of synthetic lethality and the role of c-Myc in drug resistance (263). *RAD51C*-deficient cancer cells are sensitive to the PARPI olaparib and undergo cell death by inducing G2/M cell cycle arrest and apoptosis. By contrast, silencing of *RAD51C* in resistant cancer cell lines increases the sensitivity to olaparib and decreases RAD51 foci (264).

#### 8.3.12 Loss of SLFN11

High SLFN11 expression is associated with the response to DNA-damaging agents and the overall survival of patients with colorectal and ovarian cancer (265). Conversely, SLFN11 inactivation is a determinant of PARPI resistance. Cells that express SLFN11 are more sensitive to talazoparib and olaparib than cells with low SLFN11 expression. Genomic analysis confirmed the high correlation between treatment response and SLFN11, which is considered a biomarker of the response to PARPI treatment (266). PDXs and SCLC cell lines treated with cisplatin/PARPIs show down-regulation of SLFN11 associated with therapeutic resistance. This was confirmed by silencing SLFN11, which reduced the *in vitro* sensitivity to cisplatin and PARPIs as well as drug-induced DNA damage (267). SLFN11 was identified as a relevant predictive biomarker of sensitivity to PARPI monotherapy in SCLC, and loss of SLFN11 confers resistance to PARPIs. SCLC cell lines were treated with the PARPIs olaparib, rucaparib, and veliparib, and gene expression and the HRD genomic scar score were analyzed. SLFN11 was correlated with the response to olaparib, rucaparib, and veliparib treatment but not to the HRD genomic score scar. An *in vivo* PDX model and immunohistochemical staining confirmed that loss of SLNF11 confers resistance to PARPIs (268).

#### 8.3.13 Loss of XRCC5 (Ku80) and XRCC6 (Ku70)

Loss of PARP activity leads to accumulation of SSBs, which are converted to DSBs by the cellular replication and/or transcription machinery. These DSBs can be repaired by HR in BRCA-proficient cells, whereas they accumulate in BRCA-deficient cells leading to cell death. NHEJ is initiated when free DNA ends are bound by XRCC5/Ku80 and XRCC6/Ku70 through the catalytic subunit of DNA-dependent protein kinases (DNA-PKcs). The DNA-PKc complex phosphorylates downstream targets and activates the DNA damage response, thereby initiating NHEJ (269). The NHEJ-mediated repair of DNA DSBs requires the formation of a Ku70/Ku80/DNA-PKc complex at the DSB sites. Simultaneous loss of HR and PARP1 activity results in deregulated/increased NHEJ activity, which increases the activation of DNA-PKcs leading to increased genomic instability (resulting from this error-prone pathway) (270). PARP1 plays a crucial role in suppressing NHEJ, which serves as a target of PARPI-induced lethality in HR-deficient cells. Conversely, inhibition or loss of multiple components of NHEJ such as XRCC5/Ku80, XRCC6/Ku70, and DNA-PK confer HR-deficient cells resistance to PARPIs by reducing NHEJ activity (242, 261). The activity of the error-prone NHEJ DSB repair pathway that causes genomic instability is required for PARPI sensitivity.

### 8.4 Strategies to Overcome PARP Inhibitor Resistance

Although PARPIs are likely to be beneficial for a large fraction of ovarian and breast cancer patients, the development of PARPI resistance brings challenges to their utility. As mentioned in this review, there are many mechanisms that can reverse HR deficiency to HR proficiency. Many strategies have been designed to reverse PARPI resistance (13, 105). For instance, replication fork stabilization is a compensatory mechanism for PARPI resistance. Cell cycle checkpoint (ATR, CHK1, WEE1) proteins that contribute to replication fork stabilization may be potential targets for combination therapy with PARPI by limiting the time for tumor cells to repair damaged DNA. The three proteins, ATR1, CHK1 and WEE1, play different roles in replication fork stabilization, indicating that different combination regimen may be effective for combating resistance. For example, ATR inhibitor AZD6738 sensitized *BRCA2* mutant, *BRCA2* reversion mutation, and *BRCA1* wild-type ovarian cancer cells to olaparib more effectively than the CHK1 inhibitor MK8776 (271). WEE1 may have a critical role in cell cycle arrest compared to ATR and CHK1 because WEE1 is required to maintain ATR and CHK1 activity (243). WEE1 inhibitor AZD1775 had synergistic effect with olaparib in TNBC cells (272). Even inhibition of ATR, CHK1 and WEE1 proteins effectively abrogated G2 arrest, but not sufficient to overcome PARPI resistance caused by other mechanisms such as HR pathway.

Many strategies have been designed to selectively convert HR-proficient cells to HR-deficient status. The combined use of PARPIs with CDK1 inhibitors induces HRD in HR-proficient cells by inhibiting the phosphorylation of BRCA1 by CDK1. The reduction of CDK1 compromises the capacity of cells to repair DNA using HR because BRCA1-deficient cells do not efficiently form RAD51 foci (an essential component of HRR). In addition to checkpoint activation, CDK1-mediated phosphorylation of BRCA1 is required for HR (273). The PI3K/AKT/mTOR pathway is aberrantly dysregulated in certain cancers such as TNBC; therefore, direct inhibition of the PI3K/AKT/mTOR pathway in combination with PARPIs could be an effective strategy to overcome PARPI resistance. Under normal conditions, PI3K stabilizes and conserves DSB repair by interacting with the HR complex (274). mTOR inhibitors and PARPIs show strong synergism when used in combination, as indicated by the effect of mTOR inhibitors on suppressing HRR in BRCA-proficient TNBC cell lines (275). The combined use of PARPIs with histone deacetylase inhibitors (HDIs) can sensitize cancer cells to PARPIs because HDIs block the deacetylation of heatshock protein 90 (HSP90), which leads to the degradation of several proteins such as BRCA1, RAD52, ATR, and CHK1. Direct inactivation of HSP90 is another approach to the induction of BRCAness (276, 277).

Another mechanism to induce BRCAness is the combined use of PARP and EGFR inhibitors, which alters the DSB repair capacity and activates the intrinsic pathway of apoptosis. *In vitro* and *in vivo* findings show that inhibition of EGFR1 and 2 induces a transient DNA repair deficit and alters the interaction of EGFR with BRCA1 by increasing cytosolic BRCA1 and EGFR, pulling them away from their nuclear DNA repair substrates (277, 278). Another study showed that ATM depletion sensitizes breast cancer cell lines to the PARPI olaparib (279). PARPIs in combination with androgen receptor inhibitors promote DNA damage-induced cell death, which inhibits prostate cancer cell proliferation and the growth of tumor xenografts in mice, suggesting a potentially effective treatment combination for androgen-expressing breast cancers (280).

Recently, Johnson et al. (281) reported that *BRCA*-mutant TNBC cells with acquired PARPI resistance are resensitized to PARP inhibition by dinaciclib, a potent CDK12 inhibitor that disrupts HR. In *BRCA*-mutated cancer, *de novo* resistance to PARPIs is caused by residual HR. In addition, dinaciclib compromises HR repair and sensitizes *BRCA* wild-type TBNC cells to PARP inhibition. This study also showed that dinaciclib amplifies the response to PARPIs in HR-deficient cancers. MYC inhibitors induce PARPI sensitivity. The downstream oncogenic role of MYC relies on its heterodimerization with the basic loop helix protein MAX, which is essential in causing the transcriptional initiation of targets. For instance, the small molecule 10058-F4 inhibits MYC-MAX binding (282), resulting in the supression of RAD51 in MDA-MB-231 and SUM149 cell lines (263). CDK12 inhibitor dinaciclib which downregulates MYC expression resensitizes PARPI-resistant cells to PARP inhibition when used in combination with niraparib; the synergistic effect was observed in *BRCA* wild-type and mutant TNBC cell lines in associtaion with the down-regulation of the HR gene RAD51 (263). This finding indicates that targeting the c-*Myc* oncogene could be an effective strategy to induce synthetic lethality and reverse PARPI resistance in MYC-driven cancers.

Recent preclinical and clinical studies demonstrated that the efficacy of PARPI could be enhanced in combination with immune checkpoint inhibitors (ICIs) *via* a synergistic effect. In cancers with defective DNA repair, such as HRD, accumulated DNA damage by PARPI leaded to high tumor mutational burden resulting in neoantigen formation and an increased anti-cancer immune response (283, 284). In addition, these DNA damages might increase the exposure of double-strand DNA (dsDNA) in the cytoplasm and activate the stimulator of interferon genes (STING) pathway which upregulates cytokines like type I interferon, thereby promoting immune response and recruiting tumor-infiltrating lymphocytes (TILs), especially CD8^+^ T cells (285, 286). PARPI also increases PD-L1 expression, a biomarker for ICI response, through the STING pathway (287), the ATM-ATR-CHK1 pathway (288), and inactivation of glycogen synthase kinase 3-beta (GSK3β) (289). Upregulation of PD-L1 may be a resistance mechanism of PARPI. For these reasons, subsequent immune checkpoint blockade could sensitize PARPI-treated tumor regression. Clinical trials investigating combined regimen of PARPI and ICIs such as anti-PD1 (BGB-A317, nivolumab, pembrolizumab, TSR-042), anti-PD-L1 (atezolizumab, avelumab, durvalumab), and anti-CTLA4 (ipilimumab, tremelimumab) demonstrated promising results in patient outcomes in solid tumors (290, 291).

## 9 Conclusion

HRR is the guardian of the genome because of its role in repairing DSBs with high fidelity. Defects in HR due to mutation, LOH, and promoter hypermethylation of certain HR genes result in HRD, which confers sensitivity to DNA-damaging agents and PARPIs. This review demonstrated that HRD is higher in ovarian and breast cancers than in other cancer types such as CRC, PC, NSCLC, and prostate cancer. HRD is not limited to *BRCA1* and *2*, and comprises many DNA repair genes. The fundamental vulnerability of HRD has led to the design of a wide range of HRD-directed therapies. DNA repair targeted therapies exploit DNA repair defects because HR-deficient tumors are intrinsically sensitive to PARPIs. This highlights the concept of synthetic lethality associated with the concurrent inactivation of two or more HRR genes. The use of PARPIs in non-*BRCA* mutation carriers can be expanded to sporadic cancers that display DNA repair defects. PARPIs are essential for the treatment of ovarian and breast cancers. Recently, FDA approved olaparib for the treatment of prostate and pancreatic cancers characterized by HRD. However, the benefits of PARPIs are limited by the development of resistance, especially when used as monotherapy. Many mechanisms of resistance to PARPI have been identified in HR-deficient cancers, which are challenges to overcome. Numerous preclinical and clinical studies revealed that combination therapy of PARPI with targeted chemotherapy or ICIs improved the efficacy by overcoming PARPI resistance. Understanding the mechanisms of PARPIs resistance will be useful for designing strategies to overcome PARPI resistance as summarized in this review. Based on accumulated research, more potential PARPIs and more effective combined regimens targeting HR-deficient cancers would be developed in the future.

## Author Contributions

NM, collected data, sketched the initial version, and reviewed the final manuscript. HY, reviewed and drafted the final version of the manuscript. YS, conceived the idea, guided the write up, and reviewed the final manuscript. All authors contributed to the article and approved the submitted version.

## Funding

This research was supported by a grant of the Korea Health Technology R&D Project through the Korea Health Industry Development Institute (KHIDI), funded by the Ministry of Health and Welfare, Republic of Korea (grant number: HI17C2196).

## Conflict of Interest

The authors declare that the research was conducted in the absence of any commercial or financial relationships that could be construed as a potential conflict of interest.

## Publisher’s Note

All claims expressed in this article are solely those of the authors and do not necessarily represent those of their affiliated organizations, or those of the publisher, the editors and the reviewers. Any product that may be evaluated in this article, or claim that may be made by its manufacturer, is not guaranteed or endorsed by the publisher.
