# Supplementary material for: Homologous Recombination Deficiency in Ovarian, Breast, Colorectal, Pancreatic, Non-Small Cell Lung and Prostate Cancers, and the Mechanisms of Resistance to PARP Inhibitors
Source: Front Oncol. 2022 Jun 17;12:880643. doi: 10.3389/fonc.2022.880643 (PMC9247200; doi:10.3389/fonc.2022.880643)
Supplement: Supplementary file 1 [file Table_1.docx]

Supplementary Material

# Supplementary Table S1. Characteristics and functions of DNA repair genes.

| Gene | Location in chromosome | Syndrome associated | cancer risk | Function or pathway | Ref. |
| --- | --- | --- | --- | --- | --- |
| *BRCA1* | 17q21.31 | Hereditary breast and ovarian cancer | high | Essential for DNA double strand break (DSB) repair by homologous recombination (HR) (phosphoprotein that assists in 5’ to 3’ resection of DSBs, loading of RAD51). This gene encodes a nuclear phosphoprotein that plays a role in maintaining genomic stability, and it also acts as a tumor suppressor. The encoded protein interacts with other tumor suppressors, DNA damage sensors, and signal transducers to form a large multi-subunit protein complex known as the BRCA1-associated genome surveillance complex. This protein thus plays a role in transcription, DNA repair of DSBs, and recombination. Mutations in this gene are responsible for approximately 40% of inherited breast cancers and more than 80% of inherited breast and ovarian cancers. | (1) |
| *BRCA2* | 13q13.1 | Hereditary breast and ovarian cancer | high | Essential for DNA DSB repair by HR (phosphoprotein that assists with RAD51 loading). Interacts with several other proteins to maintain breaks in DNA and also in the stability of a cell's genetic information. BRCA2 is involved in the maintenance of genome stability, specifically the homologous recombination pathway for double-strand DNA repair. | (1) |
| *NBN/*  *NBS1* | 8q21.3 | Nijmegen breakage syndrome | Moderate susceptibility | Nibrin interacts with two other proteins produced from the *MRE11A* and *RAD50* genes as part of a larger protein complex. Nibrin regulates the activity of this complex by carrying the MRE11A and RAD50 proteins into the cell's nucleus and guiding them to sites of DNA damage. The proteins work together to mend broken strands of DNA. The MRE11A/RAD50/NBN complex interacts with the protein produced from the ATM gene and recognizes broken strands of DNA and coordinates their repair. | (2) |
| *RAD50* | 5q31.1 | Nijmegen Breakage Syndrome-Like Disorder | Moderate susceptibility | The *RAD50* gene is involved in DNA DSB repair and required for both types of repair processes, NHEJ and HR. Patients with polymorphic variants and defective mutations of *RAD50* may be at a risk of developing breast cancer possibly through the loss-of-function of the MRE11-RAD50-NBN complex, which is essential for maintaining genome stability. | (3) |
| *RAD51* | 15q15.1 | Breast and ovarian cancer | Moderate susceptibility | Many studies demonstrated the association between HBOC and RAD51 paralogs (RAD51B, RAD51C, and RAD51D). RAD51 paralogs show 20%–30% amino acid sequence similarity to RAD51 and to each other. RAD51 family members are evolutionarily conserved proteins that are essential for DNA repair by HR. This protein forms a stable heterodimer with the family member RAD51C, which further interacts with other family members such as XRCC2 and XRCC3 | (4) |
| *RAD51B* | 14q24.1 | Breast and ovarian cancer | Moderate susceptibility | This protein is involved in DNA DSB repair and forms a stable heterodimer with the family member RAD51C, which further interacts with other family members such as XRCC2 and XRCC3. | (2, 4, 5) |

**Supplementary Table S1.** *Cont.*

| Gene | Location in chromosome | Syndrome associated | cancer risk | Function or pathway | Ref. |
| --- | --- | --- | --- | --- | --- |
| *RAD51C* | 17q22 | Breast and ovarian cancer | Moderate susceptibility | Involved in the HR repair pathway of DSBs, in Holliday junction replication forks, early function in DNA repair in facilitating phosphorylation of the CHEK2, protects the RAD51 complex from ubiquitin-mediated degradation and thereby transduction of the damage signal, leading to cell cycle arrest and HR activation. | (4) |
| *RAD51D* | 17q12 | Breast, ovarian and other cancers | Moderate susceptibility | Involved in the homologous recombination repair (HRR) pathway of double-stranded DNA breaks arising during DNA replication or induced by DNA-damaging agents. The protein complex formed with this protein catalyzes homologous pairing between single- and double-stranded DNA, and is thought to play a role in the early stage of recombination repair of DNA. The protein encoded by this gene is a member of the RAD51 protein family. | (4) |
| *ATM* | 11q22.3 | breast cancer/Ataxia Telangiectasia/  Pancreatic cancer | Moderate susceptibility | This protein is involved in the recognition and repair of DSBs. It encodes a protein kinase, which is a central regulator of the cellular response to DNA damage and a positive regulator of telomere lengthening. Activates different downstream proteins such as CHEK2, RAD17, RAD9, and NBN that are involved in the repair of DSBs. | (6) |
| *ATR* | 3q23 | Cutaneous telangiectasia and familial cancer syndrome/  Seckel syndrome 1 | Moderate susceptibility | DNA damage can activate the checkpoint kinase ATR for arresting S or G2 cells by inactivating the CDC25–cyclin-dependent kinase (CDK) pathway. It also activates checkpoint signaling upon genotoxic stresses, acting as a DNA damage sensor. Phosphorylates BRCA1, CHEK1, BLM, FANCD2/FANCI, RAD17, and P53, which collectively inhibit DNA replication and mitosis and promote DNA repair, recombination, and apoptosis. | (1, 7) |
| *BARD1* | 2q35 | Breast and ovarian cancer susceptibility | Moderate susceptibility | This gene encodes a protein which interacts with the N-terminal region of *BRCA1*. Its protein stable complex formation is essential for BRCA1 tumor suppression activity. The BRCA1-BARD1 heterodimer specifically mediates DNA damage repair, control of the cell cycle, ubiquitination (ubiquitin E3 ligase) and genomic stability. | (2, 8) |
| *PALB2* | 16p12.2 | Breast, ovarian, prostate and pancreatic cancer. | Moderate susceptibility | Critical in HRR through its ability to recruit BRCA2 and RAD51 to DNA breaks; binds to and co-localizes with BRCA2 in nuclear foci and permits the stable intra-nuclear localization and accumulation of BRCA2. Serves as the molecular scaffold for the formation of the BRCA1-PALB2-BRCA2 complex, which is essential for HR. Promotes the escape of BRCA2 from the effects of proteasome-mediated degradation. | (2, 3) |
| *FANCA* | 16q24.3 | Fanconi anemia, breast, ovarian cancer and many other cancer types | Moderate susceptibility | Activated in response to a certain type of DNA damage known as inter-strand cross-links (ICLs), which stops DNA replication. Core complex member; required for activation of FANCD2 and FANCI by ubiquitination. The activation of these two proteins, which bind together, attracts DNA repair proteins to the area of DNA damage to allow correction of the error and DNA replication. | (7, 9, 10) |
| *FANCD2* | 3p25.3 | Fanconi anemia, breast, ovarian cancer and many other cancer types | Moderate susceptibility | Involved in the repair of DNA DSBs, both by HR and single-strand annealing. Promotes accurate and efficient pairing of homologs during meiosis. The FANCD2 protein provides the missing link between the FA protein complex and the cellular BRCA1 repair machinery. Recruits DNA repair proteins, is mono-ubiquitylated by the FA core complex, and forms a heterodimer with FANCI. | (10, 11) |

**Supplementary Table S1.** *Cont.*

| Gene | Location in chromosome | Syndrome associated | cancer risk | Function or pathway | Ref. |
| --- | --- | --- | --- | --- | --- |
| *FANCI* | 15q25–26 | Fanconi anemia, breast, ovarian cancer and many other cancer types | Moderate susceptibility | Repair of DSBs by homologous recombination and in the repair of ICLs by promoting FANCD2 mono-ubiquitination by FANCL and participating in the recruitment to DNA repair sites. Specifically binds branched DNA: binds both single-stranded DNA (ssDNA) and double-stranded DNA (dsDNA). Participates in S phase and G2 phase checkpoint activation upon DNA damage. Forms a heterodimer with FANCD2 | (7, 10) |
| *FANCM* | 14q21.2 | Fanconi anemia, breast, ovarian cancer and many other cancer types | Moderate susceptibility | Important in FA core complex assembly, this DNA helicase is involved in the repair of Holliday junctions and replication forks. It recruits the BLM helicase during DDR. FANCM has a crucial function in the DNA damage response to ICLs. It acts as a helicase/translocase and binds adjacent to the crosslink, inducing the recruitment of the FA core complex to the site. The core complex mono-ubiquitinates the FANCI–FANCD2 complex, which triggers the accumulation of multiple nucleases and initiates the actual DNA repair processes. Depletion of FANCM affects both the FA pathway efficiency and tumorigenesis. | (7, 10, 12, 13), |
| *FANCF* | 11p14.3 | Fanconi anemia, breast, ovarian cancer, and many other cancer types | Moderate susceptibility | FANCF interacts with FANCA, FANCC, and FANCG to stabilize the dimers of FANCA, FANCC and FANCQ, which are core components of the FA DNA repair pathway. FANCF is also required for FANCD2-I ubiquitination. | (14) |
| *MRE11A* | 11q21 | Breast, ovarian and others | Moderate susceptibility | Component of the MRN complex, which plays a central role in DSB repair, DNA recombination, maintenance of telomere integrity, and meiosis. This gene encodes a nuclear protein involved in homologous recombination and forms a complex with the RAD50 homolog. By itself, the protein has 3' to 5' exonuclease activity and endonuclease activity. | (15) |
| *BRIP1* | 17q23.2 | Breast, ovarian and others | Moderate susceptibility | The protein encoded by this gene is a member of the RecQ DEAH helicase family and interacts with the BRCT repeats of breast cancer, type 1 (BRCA1). The bound complex is important in the normal DSB repair function of BRCA1. It also acts late in the Fanconi anemia pathway, after FANCD2 ubiquitination. | (8) |
| *ERCC1* | 19q13.32 | Breast cancer | - | The product of this gene functions in the nucleotide excision repair pathway and is responsible for the 5'-incision during DNA repair. It is responsible, in conjunction with SLX4, for the first step in the repair of ICLs. It is also required for homology-directed repair (HDR) of DNA DSBs in conjunction with SLX4. | (16) |
| *CHEK2* | 22q12.1 | Li–Fraumeni syndrome 2 | Moderate susceptibility | The CHEK2 gene provides instructions for the production of checkpoint kinase 2 (CHK2). This protein acts as a tumor suppressor, which means that it regulates cell division by keeping cells from growing and dividing too rapidly or in an uncontrolled way.  In response to DNA damage, the CHK2 protein interacts with several other proteins such as TP53. These proteins halt cell division and determine whether a cell will repair the damage or self-destruct in a controlled manner (undergo apoptosis). This process keeps cells with mutated or damaged DNA from dividing, which helps prevent the development of tumors. | (17) |
| *EMSY* | 11q13.5 | Breast, ovarian and others | - | The *EMSY* gene encodes a BRCA2-interacting protein that has been implicated in DNA damage repair and genomic instability. Its interaction with BRCA2 suggests that it may play a central role in the DNA repair function of BRCA2. | (18) |

**Supplementary Table S1.** *Cont.*

| Gene | Location in chromosome | Syndrome associated | cancer risk | Function or pathway | Ref. |
| --- | --- | --- | --- | --- | --- |
| *TP53* | 17p13.1 | Li–Fraumeni syndrome 1 | High penetrance | The encoded protein p53 responds to diverse cellular stresses to maintain genetic stability by inducing cell cycle arrest, apoptosis, senescence, DNA repair, or changes in metabolism. Germline mutations in TP53 are associated with hereditary cancers such as Li-Fraumeni syndrome, the predisposition to multiple cancers | (3) |
| *STK11* | 19p13.3 | Peutz–Jeghers syndrome | High penetrance | STK11 regulates cell polarity and functions as a tumor suppressor. Germline mutations in the *STK11* gene are associated with Peutz-Jegher syndrome, an autosomal dominant disorder characterized by the growth of polyps in the gastrointestinal tract, pigmented macules on the skin and mouth, and cancer susceptibility in various organs including testis, ovary, endo-cervix, breast, pancreas, and colon. | (3) |
| *PTEN* | 10q23.31 | Cowden syndrome | High penetrance | PTEN is a phosphatidylinositol 3-phosphatase that functions as a tumor suppressor by negatively regulating the AKT signaling pathway. Mutations in the *PTEN* gene are associated with the development of Cowden syndrome and correlated with hamartomatous polyps, and early-onset breast, thyroid, and endometrial cancers | (3) |
| *CDH1* | 16q22.1 | Hereditary diffuse gastric cancer/  familial lobular breast cancer | High penetrance | Involved in mechanisms regulating cell-cell adhesion. Loss of CDH1 leads to cancer progression by increasing proliferation, invasion, metastasis, and epithelial-mesenchymal transition. Germline mutations in the CDH1/E-cadherin gene predispose to the development of the autosomal hereditary  diffuse gastric cancer syndrome, which leads to the development of breast, colorectal, gastric, and ovarian cancer | (3) |
| *BLM* | 15q26.1 | Bloom syndrome | - | The BLM gene provides instructions for producing a member of the RecQ helicase family. Helicases are enzymes that attach (bind) to DNA and unwind the two spiral strands (double helix) of the DNA molecule. This unwinding is necessary for several processes including cell division and repairing damaged DNA. The BLM protein helps prevent excess sister chromatid exchanges and is also involved in processes that help maintain the stability of the DNA during the copying process. | (17) |
| *RBBP8* | 18q11.2 | Breast, ovarian and other cancers | - | Endonuclease that cooperates with the MRE11-RAD50-NBN (MRN) complex in DNA-end resection, the first step of DSB repair through the HR pathway. HR is restricted to the S and G2 phases of the cell cycle and preferentially repairs DSBs resulting from replication fork collapse. It is a key determinant of DSB repair pathway choice, as it commits cells to HR by preventing classical non-homologous end-joining (NHEJ). | (19) |
| *CDK12* | 17q12 | Breast, ovarian and other cancers | - | CDK12/cyclin K is a master regulator of proteins specifically involved in DNA damage repair (DDR) and response to DNA damage. It protects normal cells from genomic instability by regulating the transcription of DNA damage response (DDR) genes. CDK12 specifically upregulates the expression of genes involved in response to DNA damage, stress, and heat shock. Genomic alterations in CDK12 are detected in esophageal, stomach, breast, endometrial, uterine, ovarian, bladder, colorectal, and pancreatic cancers, ranging from 5% to 15% of sequenced cases. | (20) |

**Supplementary Table S1.** *Cont.*

| Gene | Location in chromosome | Syndrome associated | cancer risk | Function or pathway | Ref. |
| --- | --- | --- | --- | --- | --- |
| *TP53BP1* | 15q15.3 | Different cancers | - | This gene encodes a protein that functions in the DNA DSB repair pathway choice, promoting NHEJ pathways, and counteracting the function of the HR repair protein BRCA1. This protein plays multiple roles in the DNA damage response, including promoting checkpoint signaling following DNA damage, acting as a scaffold for recruitment of DNA damage response proteins to damaged chromatin, and promoting NHEJ pathways by limiting end resection following a DSB. | (21) |
| *XRCC1* | 19q13.31 | - | - | The protein encoded by this gene is involved in the efficient repair of DNA single-strand breaks formed by exposure to ionizing radiation and alkylating agents. This protein interacts with DNA ligase III, polymerase beta, and poly (ADP-ribose) polymerase to participate in the base excision repair pathway. | (22) |
| *MAD2L2/REV7* | 1p36.22 | - | - | A component of the mitotic spindle assembly checkpoint that prevents the onset of anaphase until all chromosomes are properly aligned at the metaphase plate. Component of the shieldin complex, which plays an important role in repair of DNA DSBs. During G1 and S phases of the cell cycle, the complex functions downstream of TP53BP1 to promote NHEJ and suppress DNA end resection. Mediates various NHEJ-dependent processes including immunoglobulin class-switch recombination, and fusion of unprotected telomeres. | (23) |
| *XRCC5/*  *Ku80* | 2q35 | - | - | Important in NHEJ DNA repair, the Ku70/Ku80 heterodimer forms a ring around DNA break ends and then recruits DNA-dependent protein kinase catalytic subunit (DNA-PKcs) to complete the ligation and joining of DNA breaks. The Ku70/Ku80/DNA-PKcs complex forms a trimer at DSB sites, a prerequisite for NHEJ-mediated repair in mammalian cells. The regulation of complex formation could affect the NHEJ activity and subsequently the therapeutic sensitivity. The N-termini of Ku70/80 share sequence and structural similarity and the homologs of the Ku proteins form a heterodimer that binds with high affinity to double-stranded DNA. | (24) |
| *XRCC6/*  *Ku70* | 22q13.2 | - | - | Important in NHEJ DNA repair, the Ku70/Ku80 heterodimer forms a ring around DNA break ends and then recruits DNA-PKcs to complete the ligation and joining of DNA breaks. The c-terminus of Ku70 interacts with DNA as well as transcription factors involved in different processes. | (24) |
| *SLFN11* | 17q12 | - | - | SLFN11 is actively recruited to sites of DNA damage to inhibit HR and is important in PARPI sensitivity. SLFN11 binds replication forks in response to replication stress, blocks replication regardless of ATR-CHK1 activity, opens chromatin in the near vicinity of replication initiation sites, kill cells with defective replication and serves as a guardian of the genome | (25) |

**References**

1. Rimar KJ, Tran PT, Matulewicz RS, Hussain M, Meeks JJ. The emerging role of homologous recombination repair and PARP inhibitors in genitourinary malignancies. Cancer. 2017;123(11):1912-24.

2. Siraj AK, Masoodi T, Bu R, Parvathareddy SK, Al-Badawi IA, Al-Sanea N, et al. Expanding the spectrum of germline variants in cancer. Human genetics. 2017;136(11-12):1431-44.

3. Kobayashi H, Ohno S, Sasaki Y, Matsuura M. Hereditary breast and ovarian cancer susceptibility genes (review). Oncology reports. 2013;30(3):1019-29.

4. Sanchez-Bermudez AI, Sarabia-Meseguer MD, Garcia-Aliaga A, Marin-Vera M, Macias-Cerrolaza JA, Henarejos PS, et al. Mutational analysis of RAD51C and RAD51D genes in hereditary breast and ovarian cancer families from Murcia (southeastern Spain). European journal of medical genetics. 2018;61(6):355-61.

5. Wadt KAW, Aoude LG, Golmard L, Hansen TVO, Sastre-Garau X, Hayward NK, et al. Germline RAD51B truncating mutation in a family with cutaneous melanoma. 2015;14(2):337-40.

6. Renault AL, Mebirouk N, Cavaciuti E, Le Gal D, Lecarpentier J, d'Enghien CD, et al. Telomere length, ATM mutation status and cancer risk in Ataxia-Telangiectasia families. Carcinogenesis. 2017;38(10):994-1003.

7. Quezada Urban R, Díaz Velásquez CE, Gitler R, Rojo Castillo MP, Sirota Toporek M, Figueroa Morales A, et al. Comprehensive Analysis of Germline Variants in Mexican Patients with Hereditary Breast and Ovarian Cancer Susceptibility. Cancers. 2018;10(10).

8. Schoolmeester JK, Moyer AM, Goodenberger ML, Keeney GL, Carter JM, Bakkum-Gamez JN. Pathologic findings in breast, fallopian tube, and ovary specimens in non-BRCA hereditary breast and/or ovarian cancer syndromes: a study of 18 patients with deleterious germline mutations in RAD51C, BARD1, BRIP1, PALB2, MUTYH, or CHEK2. Human pathology. 2017;70:14-26.

9. Chatterjee A, Rodger EJ, Eccles MR. Epigenetic drivers of tumourigenesis and cancer metastasis. Semin Cancer Biol. 2018;51:149-59.

10. Nalepa G, Clapp DW. Fanconi anaemia and cancer: an intricate relationship. Nature reviews Cancer. 2018;18(3):168-85.

11. Houghtaling S, Newell A, Akkari Y, Taniguchi T, Olson S, Grompe M. Fancd2 functions in a double strand break repair pathway that is distinct from non-homologous end joining. Human molecular genetics. 2005;14(20):3027-33.

12. Bogliolo M, Bluteau D, Lespinasse J, Pujol R, Vasquez N, d'Enghien CD, et al. Biallelic truncating FANCM mutations cause early-onset cancer but not Fanconi anemia. Genetics in medicine : official journal of the American College of Medical Genetics. 2018;20(4):458-63.

13. Khiabanian H, Hirshfield KM, Goldfinger M, Bird S, Stein M, Aisner J, et al. Inference of Germline Mutational Status and Evaluation of Loss of Heterozygosity in High-Depth, Tumor-Only Sequencing Data. JCO precision oncology. 2018;2018.

14. D'Andrea AD. Susceptibility pathways in Fanconi's anemia and breast cancer. The New England journal of medicine. 2010;362(20):1909-19.

15. Brandt S, Samartzis EP, Zimmermann AK, Fink D, Moch H, Noske A, et al. Lack of MRE11-RAD50-NBS1 (MRN) complex detection occurs frequently in low-grade epithelial ovarian cancer. BMC cancer. 2017;17(1):44.

16. Li B, Shi X, Yuan Y, Peng M, Jin H, Qin D. ERCC1 rs11615 polymorphism increases susceptibility to breast cancer: a meta-analysis of 4547 individuals. Bioscience reports. 2018;38(3).

17. Sokolenko AP, Bogdanova N, Kluzniak W, Preobrazhenskaya EV, Kuligina ES, Iyevleva AG, et al. Double heterozygotes among breast cancer patients analyzed for BRCA1, CHEK2, ATM, NBN/NBS1, and BLM germ-line mutations. Breast cancer research and treatment. 2014;145(2):553-62.

18. Määttä KM, Nurminen R, Kankuri-Tammilehto M, Kallioniemi A, Laasanen S-L, Schleutker J. Germline EMSY sequence alterations in hereditary breast cancer and ovarian cancer families. BMC cancer. 2017;17(1):496.

19. TCGA. Integrated genomic analyses of ovarian carcinoma. Nature. 2011;474(7353):609-15.

20. Lui GYL, Grandori C, Kemp CJ. CDK12: an emerging therapeutic target for cancer. Journal of Clinical Pathology. 2018;71(11):957-62.

21. TCGA. Comprehensive molecular portraits of human breast tumours. Nature. 2012;490(7418):61-70.

22. Liu GC, Zhou YF, Su XC, Zhang J. Interaction between TP53 and XRCC1 increases susceptibility to cervical cancer development: a case control study. BMC cancer. 2019;19(1):24.

23. Noordermeer SM, Adam S, Setiaputra D, Barazas M, Pettitt SJ, Ling AK, et al. The shieldin complex mediates 53BP1-dependent DNA repair. Nature. 2018;560(7716):117-21.

24. Scott CL, Swisher EM, Kaufmann SH. Poly (ADP-ribose) polymerase inhibitors: recent advances and future development. Journal of clinical oncology : official journal of the American Society of Clinical Oncology. 2015;33(12):1397-406.

25. Murai J, Tang S-W, Leo E, Baechler SA, Redon CE, Zhang H, et al. SLFN11 Blocks Stressed Replication Forks Independently of ATR. Molecular Cell. 2018;69(3):371-84.e6.
